# QBO deepens MJO convection

**DOI:** 10.1038/s41467-023-39465-7

**Published:** 2023-07-10

**Authors:** Daeho Jin, Daehyun Kim, Seok-Woo Son, Lazaros Oreopoulos

**Affiliations:** 1 University of Maryland - Baltimore County, Baltimore, MD USA; 2 Earth Sciences Division, NASA’s Goddard Space Flight Center, Greenbelt, MD USA; 3 University of Washington, Seattle, WA USA; 4 Seoul National University, Seoul, South Korea

**Keywords:** Atmospheric dynamics, Climate and Earth system modelling

## Abstract

The underlying mechanism that couples the Quasi-Biennial Oscillation (QBO) and the Madden-Julian oscillation (MJO) has remained elusive, challenging our understanding of both phenomena. A popular hypothesis about the QBO-MJO connection is that the vertical extent of MJO convection is strongly modulated by the QBO. However, this hypothesis has not been verified observationally. Here we show that the cloud-top pressure and brightness temperature of deep convection and anvil clouds are systematically lower in the easterly QBO (EQBO) winters than in the westerly QBO (WQBO) winters, indicating that the vertical growth of deep convective systems within MJO envelopes is facilitated by the EQBO mean state. Moreover, the deeper clouds during EQBO winters are more effective at reducing longwave radiation escaping to space and thereby enhancing longwave cloud-radiative feedback within MJO envelopes. Our results provide robust observational evidence of the enhanced MJO activity during EQBO winters by mean state changes induced by the QBO.

## Abstract (web summary)

This paper shows that the vertical growth of deep convective systems within Madden-Julian oscillation envelopes is facilitated by mean state changes in the upper-troposphere and lower-stratosphere during easterly Quasi-Biennial Oscillation winters.

## Introduction

The Madden-Julian Oscillation (MJO)^1,2^, the dominant mode of tropical intraseasonal variability during boreal winter, is distinguishable from other modes of organized tropical convection by its vast horizontal scale (wavenumber 1–6), characteristic quasi-periodicity of 30–60 days, and slow eastward propagation speed of about 5 ms^−1^ over the Indo-Pacific warm pool. By modulating circulation anomalies globally, the MJO affects many Earth system phenomena, including the formation and tracks of tropical cyclones^3,4^, the onsets and breaks of the Asian and Australian summer monsoons^5^, the North Atlantic oscillation^6^, and Arctic sea ice variability^7^, to name a few. The MJO is also a known driver of many types of extreme events all over the globe^8^, such as extreme rainfall events^9^, flooding^10^, cold surges^11^, fires^12^, lightning^13^, and tornados^14^. Given the MJO’s bold fingerprint on the location, frequency, and intensity of extreme events, a realistic representation of the MJO is arguably a prerequisite for any numerical weather and climate model to accurately simulate and predict societally relevant extreme events. However, the maintenance and propagation mechanism of the MJO has not been fully understood^15,16^, and many global climate models still represent it poorly^17^.

A roadblock in the quest towards a complete understanding of the MJO is its relationship with the Quasi-Biennial Oscillation (QBO), a leading mode of interannual variability in the equatorial stratosphere characterized by alternating westerly and easterly zonal-mean zonal wind with a periodicity of about 20–30 months^18–20^. While the QBO’s influence on tropical convection had been reported previously^21–23^, its strong modulation of MJO variability was reported only recently^24–28^. For example, MJO activity is more pronounced across the Indo-Pacific warm pool region, and the propagation of the MJO is disrupted less frequently in the Maritime Continent (MC) region during easterly QBO (EQBO) winters (December-February, DJF) than during westerly QBO (WQBO) winters. Despite the robust observational evidence of a QBO-MJO relationship, the underlying mechanism has remained elusive^29^, with many pertinent questions, such as why a strong QBO-MJO connection appears only in DJF, remaining unanswered. Furthermore, no existing Global Climate Model (GCM) realistically reproduces the observed QBO-MJO relationship^30–32^, even when a QBO signal is prescribed through nudging^33^.

A popular idea for the observed QBO-MJO connection is the QBO temperature stratification mechanism^24,25,28,29,34–36^. For example, Hendon and Abhik^28^ argued that the zonal-mean temperature anomalies associated with QBO affect the MJO by modulating the strength of the cold cap (the layer of anomalously negative temperature perturbations at around 100-hPa) above the MJO convection. They found that the cold cap above the enhanced convection associated with the MJO is much stronger during EQBO winters, possibly due to the amplified Kelvin wave response^37,38^. Combined with the positive temperature anomalies beneath it, the cold cap destabilizes the upper troposphere and lower stratosphere (UTLS), and provides a favorable condition for convection growth and maintenance.

The QBO-MJO relationship is likely determined by not only adiabatic but also diabatic processes such as longwave (LW) heating of the atmospheric column from cloud-radiation interactions^25,35^. Modeling and observational evidence suggests that LW cloud-radiation feedback is a crucial maintenance mechanism of the MJO^39–43^, influencing its spatial scale^44^, overall strength^45^ and the ability of the MJO to survive the Maritime Continent barrier effect^46^, among other characteristics. Sakaeda et al.^35^ suggested that the MJO may be uniquely affected by QBO-related upper tropospheric stability changes due to its sensitivity to LW cloud-radiation feedback.

Considering that the upper tropospheric temperature and static stability changes associated with QBO can directly affect the properties of anvil clouds, such as their horizontal extent^47^, the following hypothesis is put forth: the convective clouds within MJO envelopes strengthen and grow further vertically under the influence of EQBO; they are accompanied by a greater amount of anvil clouds at higher altitudes, which amplifies their radiative feedback; and the stronger radiative feedback in turn contributes to the enhanced MJO activity. While partially demonstrated in idealized model simulations^36,48^, this sequence of processes has not been verified with observations. In particular, the changes in the properties of convective clouds associated with the QBO state have not been characterized observationally. While the lower static stability in the UTLS would ease the vertical movement of air parcels and provide a favorable condition for deep convection, it is unclear whether the observed static stability change during QBO winters alters sufficiently the depth and strength of MJO convection.

The main goal of this study is therefore to assess the extent to which the properties of deep convective clouds within MJO envelopes are altered by the QBO state, with a specific focus on the vertical extent and radiative properties of deep cumulonimbus and anvil clouds. We combine multiple satellite products and employ the Cloud Regime concept^49,50^ to derive these cloud properties in an objective manner (see Methods). We show that the tops of deep convection and anvil clouds reach higher altitudes, and those clouds affect the atmospheric radiation budget more strongly so as to invigorate the MJO more during EQBO than WQBO winters.

## Results

### QBO-induced mean state changes in the Maritime Continent area

**Fig. 1:**
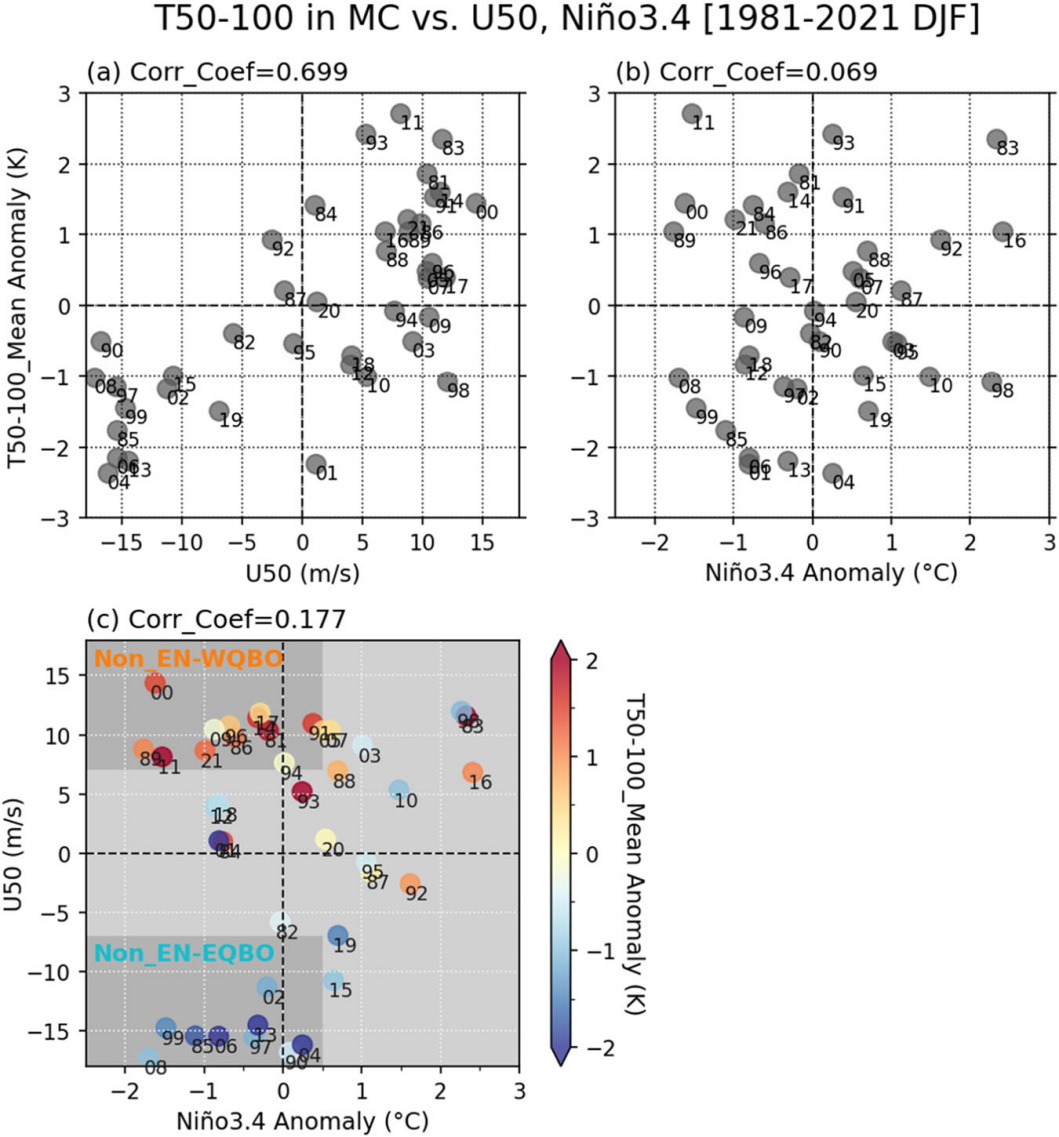
Selecting the westerly Quasi-Biennial Oscillation (WQBO) and easterly QBO (EQBO) years. Domain mean temperature in the Maritime Continent (MC; 100°E−150°E, 15°S−5°N) for layers between 50 and 100 hPa (T50-100) is aligned with (**a**) zonal mean zonal wind at 50-hPa (U50), and (**b**) Niño 3.4. Panel (**c**) shows a scatter plot of U50 vs. Niño3.4, and the level of T50-100 is presented in colors. All values are wintertime mean anomalies for December to February (DJF), and the correlation coefficient between variables of *x* and *y* axes is shown above each panel. The number next to each circle symbol indicates the last two digits of the year to which the particular January belongs to. In panel (**c**), the darker gray color areas indicate the WQBO and EQBO winters used in this study.

We first examine the mean state changes associated with the QBO in the MC region during boreal winter, when MJO activity is most sensitive to QBO state^25^ (Fig. 1). The corresponding results for the Indian Ocean (IO) and West Pacific (WP) basins are similar, although temperature perturbations are slightly weaker in the IO (not shown; see also Figs. S1 and 8). Figure 1a-b show 50-100 hPa temperature (T50-100) anomalies in the region as a function of the QBO (Fig. 1a) and El Niño-Southern Oscillation (ENSO, Fig. 1b) states. Hendon and Abhik^28^ suggested that QBO temperature anomalies at these levels either constructively or destructively interfere with the cold cap above MJO convection, affecting whether the convection further strengthens or weakens. While T50-100 anomalies show a strong positive correlation with the QBO phase (Fig. 1a), as expected, they are also weakly affected by the ENSO phase (Fig. 1b). When both ENSO and QBO are considered (Fig. 1c), the contrast in T50-100 between EQBO and WQBO winters is strong during the La Niña and ENSO-neutral winters. In the years with a pronounced El Niño signal (Niño 3.4 > 1), however, T50-100 anomalies appear to be similar between EQBO and WQBO winters (Fig. 1c). Moreover, an EQBO-WQBO comparison cannot be made for extreme El Niño winters (Niño 3.4 > 2) because none of them are in EQBO state. This result suggests that the effect of QBO-associated changes in T50-100 can be better investigated outside of El Niño winters. In the following, we focus on the contrast between EQBO and WQBO during non-El Niño (Niño 3.4 < 0.5) winters (Table 1).

**Table 1:** Selected periods for Quasi-Biennial Oscillation composites indicated by year numbers (e.g., 2005 indicates 2004 December to 2005 February)

	Non-El Niño (La Niña + Neutral; Niño 3.4 < 0.5)
WQBO	1981, 1986, 1989, 1991, 1994, 1996, 2000, **2009, 2011, 2014, 2017, 2021**
EQBO	1985, 1990, 1997, 1999, 2002, **2004, 2006, 2008, 2013**
Neutral QBO	1982, 1984, 1993, 2001, **2012, 2018**

The years in bold are those for which satellite cloud observations are available.

**Fig. 2:**
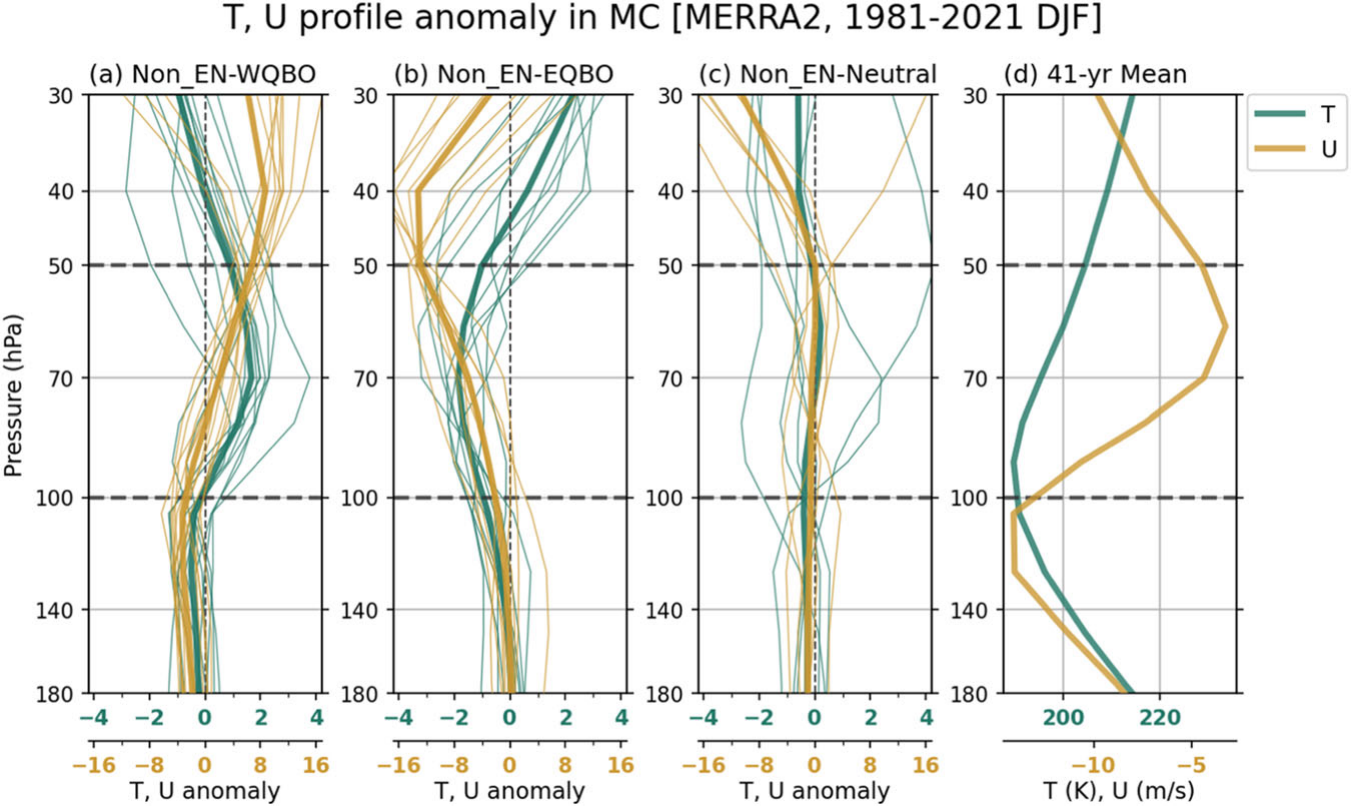
Quasi-Biennial Oscillation (QBO)-associated temperature and wind perturbations in the upper troposphere and lower stratosphere. December to February (DJF) mean seasonal anomalies of air temperature (T; green) and zonal wind (U; brown) averaged over Maritime Continent (MC; 100°E−150°E, 15°S−5°N) composited for (**a**) westerly QBO (WQBO), (**b**) easterly QBO (EQBO), and (**c**) QBO-neutral states during Non-El Niño winters. Panel (**d**) shows the 41-year climatological DJF mean of T and U profiles (1981–2021). The think lines indicate the averages of the selected years (listed in Table 1).

Figure 2 shows temperature and zonal wind anomalies in the MC region during WQBO (Fig. 2a), EQBO (Fig. 2b), and QBO-neutral (Fig. 2c) winters. Again, the temperature and wind anomalies are qualitatively similar in the IO and WP regions (not shown). The MC region experiences relatively larger changes in the magnitude and vertical extent of QBO-related temperature anomalies than the other regions, especially in EQBO winters (Fig. S1). During WQBO winters, positive temperature anomalies appear from 40 to 100 hPa, mostly due to the thermal wind balance (Fig. 2a). In contrast, in EQBO winters, negative temperature anomalies prevail in the layers around 70 hPa, which extend down below 100 hPa (Fig. 2b). Note that Nie and Sobel^48^ showed that for the QBO-temperature perturbations to alter the depth of deep convection they need to penetrate down enough, below the troposphere.

Considering that the minimum temperature appears near 90 hPa, right above the cold-point tropopause^37^ in the climatological mean (Fig. 2d), the negative temperature anomalies under the EQBO state would weaken the static stability around the tropopause, providing a favorable condition for convective cloud deepening^28^. On the other hand, the positive temperature anomalies in WQBO winters would lower the tropopause and enhance static stability above it, obstructing the vertical growth of convection. Note that using the shorter period of available satellite cloud observations (see Methods) gives a consistent result (Fig. S2).

While plausible, it is not obvious that such temperature and associated static stability changes are large enough to yield significant changes in convective cloud properties, especially cloud top height. We present below evidence from observations of convective cloud characteristics that the depth of convective clouds within MJO envelopes does indeed respond systematically in a way that is consistent with the temperature and static stability changes.

### Modulations of the large-scale MJO envelop by the QBO

**Fig. 3:**
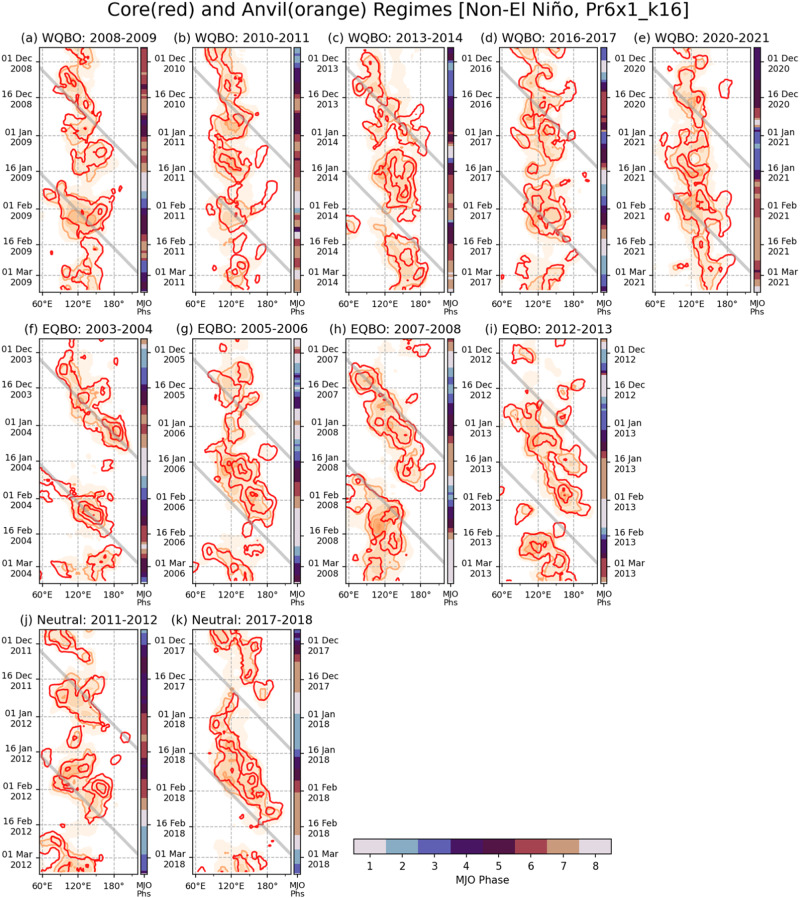
Madden-Julian Oscillation (MJO) propagation characteristics. Temporal evolutions of meridional (15°S−5°N) mean density of convective core (red contour) and anvil regimes (orange shading), subjected to 15-degree longitude and 5-day running mean filtering, for (**a**–**e**) the westerly Quasi-Biennial Oscillation (WQBO), (**f**–**i**) the easterly QBO (EQBO), and (**j**–**k**) QBO-neutral states during Non-El Niño winters. Contour interval is 10% in mean relative frequency of occurrence (RFO), and the diagonal gray lines indicate 5 m s^−1^ propagation speed. The vertical color bar next to each panel indicates the MJO phase for each day.

Before examining properties of individual deep convective clouds, we check whether the cloud observations give results that are consistent with what has been previously reported in the literature, namely enhanced eastward propagation of MJO convection across the MC region during EQBO winters compared to WQBO and QBO-neutral winters. Figure 3 shows Hovmöller diagrams of the relative frequency of occurrence (RFO) of the convective Core and Anvil regimes, which are indicative of deep convective systems (see Methods), for WQBO (top), EQBO (middle), and QBO-neutral (bottom) winters. It is apparent that the deep convective systems in EQBO winters show strong tendency of eastward propagation. Although there are just four EQBO winters, a smooth progression of the large-scale MJO envelopes across the MC to the WP region is observed in all cases. In contrast, the Core and Anvil regime RFOs in WQBO winters are mostly stagnant near the MC region. The only exception is the 2013–14 season (Fig. 3c), in which a weak eastward propagation of the MJO envelope is observed in the early period. The two QBO-neutral winters can be characterized by intermittent development and decay of large-scale convective systems, which stall (Jan–Feb 2012), or propagate either to the east (Jan–Feb 2018) or west (Dec 2017). Our results based on the Core and Anvil regimes are consistent with findings in previous studies^25^, lending confidence to the cloud classification we employ to investigate convective cloud properties.

**Fig. 4:**
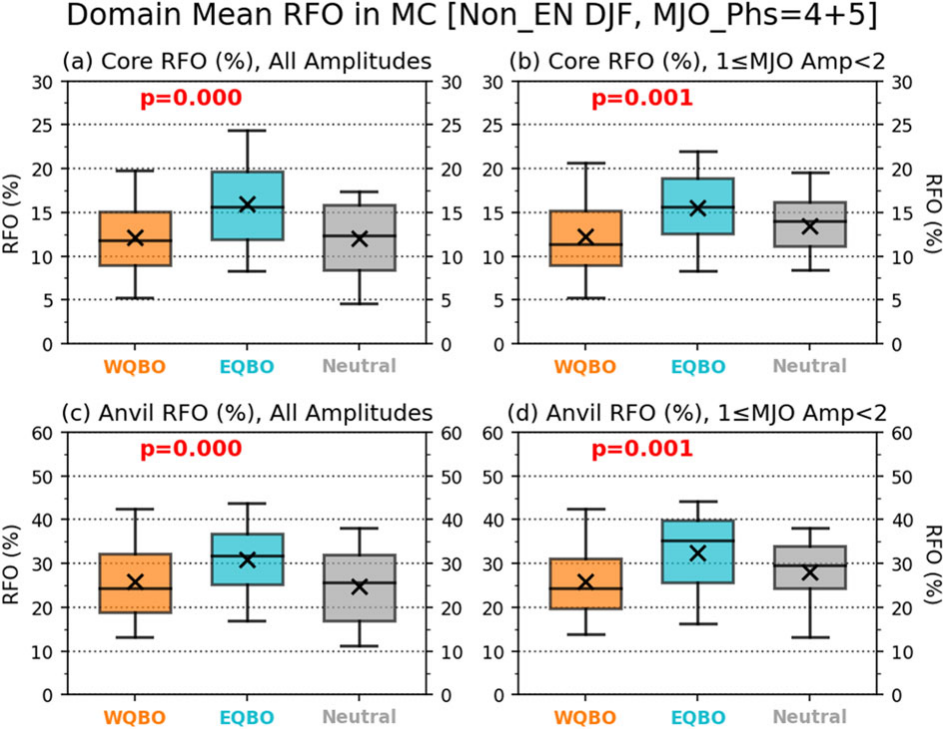
Enhanced convective activity over the Maritime Continent (MC) during easterly Quasi-Biennial Oscillation (EQBO) winters. Relative frequency of occurrence (RFO) distributions of (**a**, **b**) Core regimes (regimes 1 and 2) and (**c**, **d**) Anvil regimes (regimes 4 and 6) displayed as box-whisker plots, separated for all Madden-Julian Oscillation (MJO) amplitudes (left column) and MJO amplitudes between 1 and 2 (right column), in the MC domain (100°E−150°E, 15°S−5°N) and for days of MJO phases 4 or 5 during Non-El Niño winters (December–February). The vertical widths of boxes represent interquartile range, whiskers extend from 5% to 95%, and horizontal lines and x symbols indicate median and mean values, respectively. The p-values from t-test of westerly QBO (WQBO) and EQBO composite differences are shown in each panel. Sample sizes are 128 (WQBO) and 82 days (EQBO) for the all-amplitudes case, and 59 and 44 days for the case of MJO amplitude between 1 and 2.

Figure 3 suggests that EQBO state may help propagate MJO eastward across the MC region and reach the WP region by promoting convective activity in those regions. In Fig. 4, we compare the RFO of the Core and Anvil regimes in the MC region between WQBO, EQBO and QBO-neutral winters for MJO phases 4 and 5, in which the main MJO envelope occurs in the MC region (Fig. S3). Figure 4a, c shows that the mean RFO of the Core and Anvil regimes increases from 12.2% to 15.9%, and from 25.8% to 31.0%, respectively, in EQBO winters (blue) compared to WQBO winters (orange). The differences in the RFOs are robust with *p*-values <0.005. The RFO values in the QBO-neutral winters are in between those of the EQBO and WQBO winters. Therefore, our results show that the enhanced MJO activity during EQBO winters is associated with the increase in Core and Anvil regime clouds. The Core and Anvil regime RFOs are fairly correlated with the MJO amplitude (measured by the Real-time Multivariate MJO, see Methods) in the MC region (Fig. S4), as well as in the WP region (Fig. S5). Nonetheless, the EQBO vs. WQBO contrasts remain similar when only days with moderate MJO amplitude (between 1 and 2) are used (Fig. 4b, d).

### QBO deepens MJO convection

The results shown so far indicate a systematic increase in the occurrences of deep convective systems in the MC and WP regions in the EQBO winters. Demonstrating a causality from this correlation requires observations of robust changes in the properties of deep convective clouds that are consistent with the EQBO-related negative temperature anomalies in the UTLS (i.e., a deepening of the deep convective clouds).

**Fig. 5:**
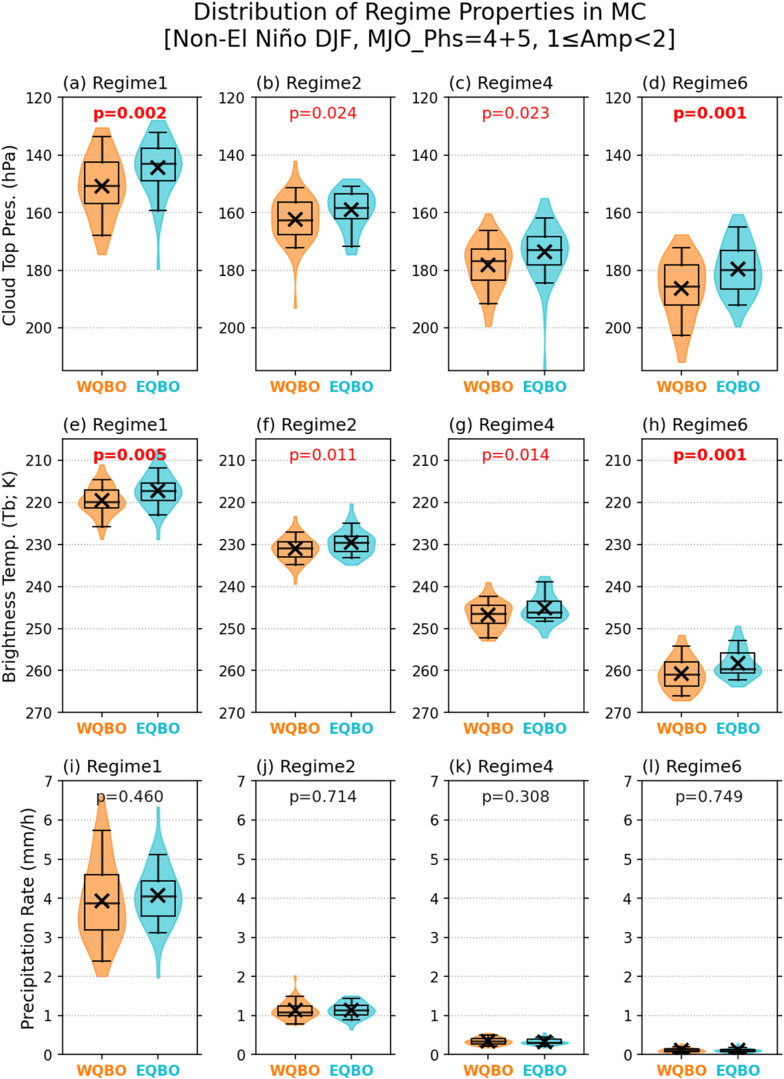
Comparison of cloud top pressure, brightness temperature, and precipitation in deep convective systems within Madden-Julian oscillation (MJO) envelops between easterly Quasi-Biennial Oscillation (EQBO) and westerly QBO (WQBO) winters. Distributions of Maritime Continent (100°E−150°E, 15°S−5°N) domain mean cloud top pressure (top row) and brightness temperature (middle row), and precipitation rate (bottom row) of grid cells identified as regimes (**a**, **e**, **i**) regime 1, (**b**, **f**, **j**) regime 2, (**c**, **g**, **k**) regime 4, and (**d**, **h**, **l**) regime 6, for westerly Quasi-Biennial Oscillation (WQBO; orange) and EQBO (blue) composite days, in violin-style box-whisker plot (same convention as in Fig. 4). The conditions for compositing are simultaneous occurrence of MJO phases 4 or 5, and MJO amplitude in the 1–2 range during Non-El Niño winters. Total sample sizes for WQBO and EQBO years satisfying these conditions are 59 and 44 days, respectively. The significance of mean difference between WQBO and EQBO composites are obtained via a *t*-test, with the corresponding p-value shown at the top of each panel.

Figure 5 shows the distributions of cloud top pressure (CTP), brightness temperature (BT), and precipitation rate of the Core and Anvil regime grid cells in the MC region, for the WQBO and EQBO winters. As in Figs. 4b, d, only the days with MJO amplitude between 1 and 2 are used. The CTP and BT are obtained as the average values of the pixels within each 1-deg grid cell (see Methods). For all Core and Anvil regimes, CTP and BT are systematically lower during EQBO vs. WQBO winters, and the differences are statistically significant at the 97.5% or higher confidence levels. Similar results are found in the WP region (Fig. S7), although the confidence levels are overall lower. In the IO, on the other hand, CTP and BT differences between EQBO and WQBO winters are not statistically significant at the 95% confidence level, except for the CTP of regime 2 (Fig. S8).

**Fig. 6:**
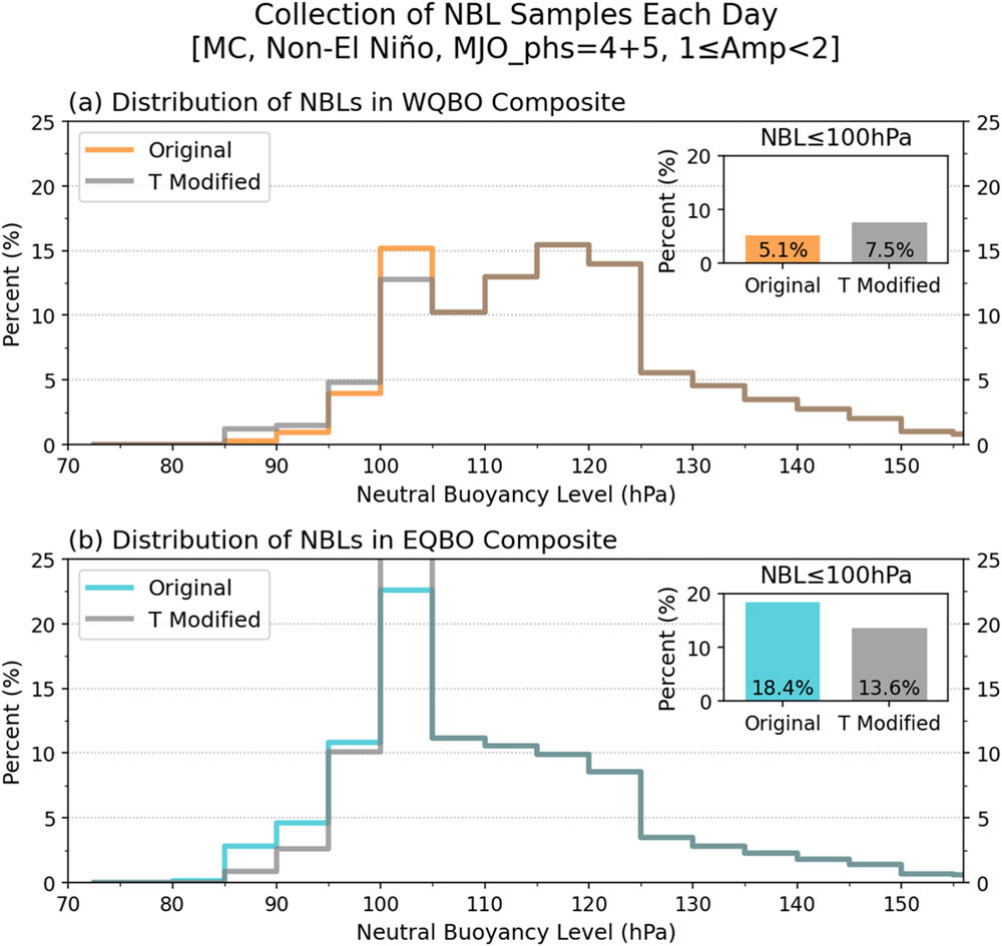
Impacts of Quasi-Biennial Oscillation (QBO)-associated temperature anomalies in the upper troposphere and lower stratosphere on the neutral buoyancy level (NBL) of non-entraining plumes. Distributions of NBLs in the Maritime Continent (MC; 100°E−150°E, 15°S−5°N) domain for (**a**) westerly QBO (WQBO; orange) and (**b**) easterly QBO (EQBO; light blue) composite days. The Madden-Julian Oscillation (MJO) and El Niño-Southern Oscillation (ENSO) conditions for compositing are same to those in Fig. 5. The gray lines labeled T Modified show the distribution of NBL when the mean temperature difference of upper atmosphere (above 100 hPa) between EQBO and WQBO is either added to WQBO profiles (panel **a**) or subtracted from EQBO profiles (panel **b**). The small insert in each panel shows the percentage of NBL ≤100-hPa. The percentage values on the *y*-axis indicate the relative population to the total number of grid cells in the MC domain during the WQBO and EQBO composite days.

The results shown in Figs. 5 and S7 suggest that the vertical growth of deep convective systems within MJO envelopes is facilitated by the EQBO state, most likely through weakened thermal stratification in the upper troposphere. To further test this, we examined the neutral buoyancy levels (NBLs; a.k.a. equilibrium level) of hypothetical convective parcels and their responses to the temperature changes associated with QBO (Fig. 6). The NBL was obtained using a non-entraining, pseudo-adiabatic plume model as the maximum possible neutral buoyancy level of the near surface parcel (see Methods) in each MERRA-2 grid in the MC region. Under the same MJO conditions used in Fig. 5, convective plumes whose NBL are lower than 100-hPa pressure level (roughly about 16-km in altitude) are found in about 5.1% and 18.4% of all grid cells considered under the WQBO and EQBO conditions, respectively. Then the same calculation is repeated after altering temperature above 100-hPa by subtracting the seasonal mean EQBO-WQBO temperature difference from the EQBO grid cells and adding the same temperature perturbations to the WQBO grid cells. When the mean temperature difference above 100-hPa is subtracted from the EQBO grid cells, the EQBO-WQBO difference in the frequency of NBLs lower than 100-hPa is reduced from 13.3% (18.4% minus 5.1%) to 8.5%, a 36% decrease. Adding the temperature difference to the WQBO grid cells also deceases the difference between EQBO and WQBO winters in the fraction of NBL values lower than 100-hPa by about 18% (from 13.3% to 10.9%). This result suggests that the tallest convective plumes are affected by the temperature anomalies associated with QBO.

**Fig. 7:**
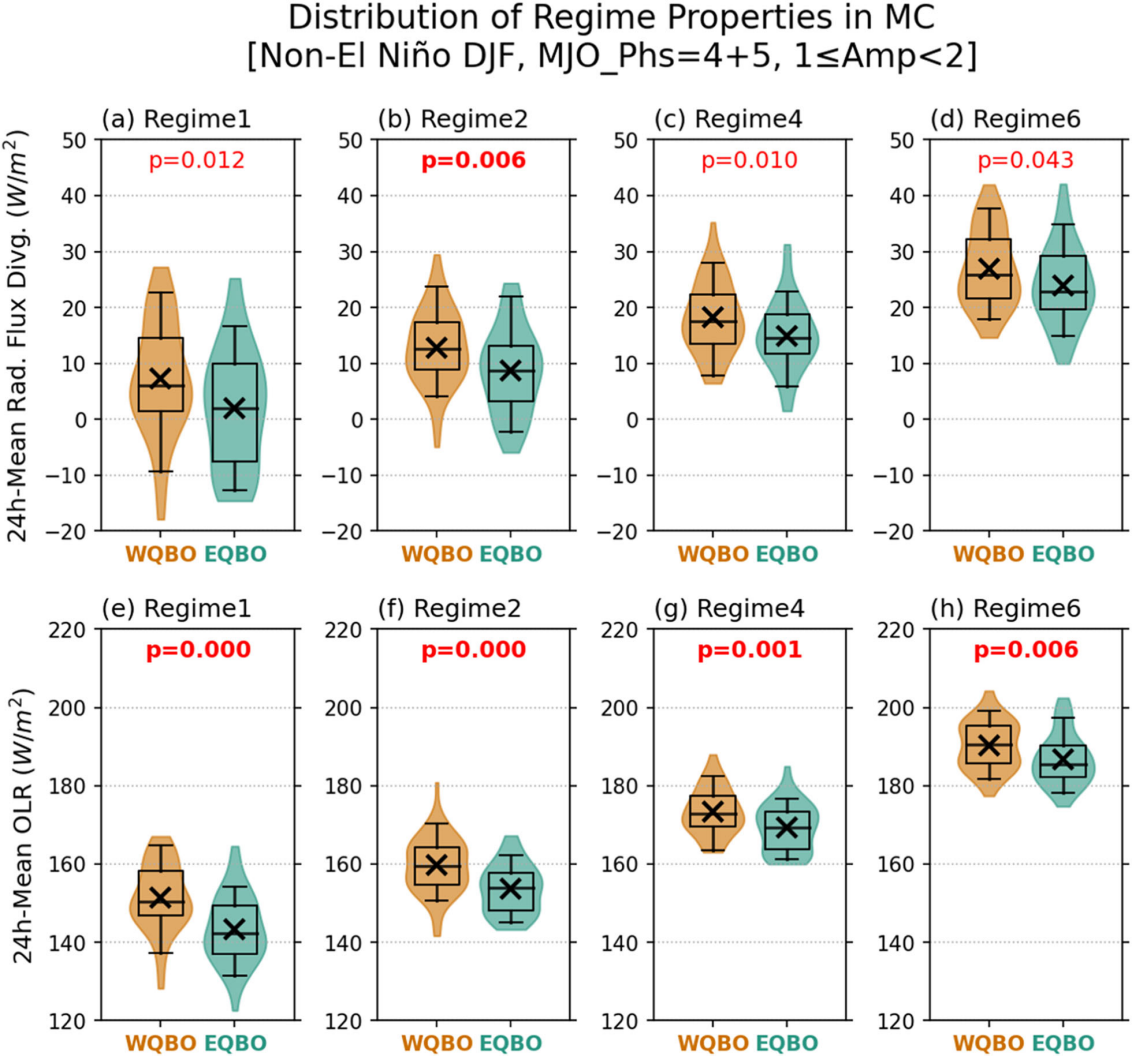
Comparison of column radiative flux convergence and outgoing longwave radiation (OLR) in deep convective systems within Madden-Julian oscillation (MJO) envelops between easterly Quasi-Biennial Oscillation (EQBO) and westerly QBO (WQBO) winters. As Fig. 5, but for (**a**–**d**) 24-h mean total (shortwave+longwave) radiative flux divergence and (**e**–**h**) OLR. Positive values indicate a loss of energy from atmospheric column.

While it has been suggested that the taller deep convective systems help better maintain MJO envelopes during EQBO winters via strengthening the LW cloud-radiation feedback^25, 35,51^, atmospheric radiation budget within deep convective systems have not been shown in the literature. Figure 7 compares 24h-mean CERES column total radiative flux divergence and outgoing LW radiation (OLR) for all Core and Anvil regime grid cells between WQBO and EQBO winters for the MC region. Positive values indicate a loss of energy from the atmospheric column for both quantities. Even with deep cumulonimbus and anvil clouds, the atmosphere mostly loses energy through the radiative processes (top row) except for some fraction of regime 1 and 2 grid cells, in which the OLR is reduced enough for the net flux divergence to be negative, i.e., radiative energy gain for the atmospheric column (Fig. 7e, f). For all Core and Anvil regimes, OLR is reduced in EQBO winters more than in WQBO winters, lowering the atmospheric column’s radiative flux divergence (i.e., increasing retained radiative energy in the column; see Fig. S12 for more details). This result indicates that the convective systems developed within MJO envelopes in EQBO winters are more effective at reducing the longwave radiation escaping to space (i.e., a stronger decrease in effective emission temperature).

It is worthwhile to note that the differences in grid-mean precipitation are statistically insignificant in the MC region (Fig. 5i–l), suggesting that while individual deep convective clouds tend to be taller, they produce similar amounts of precipitation. This further suggests stronger LW cloud-radiation feedback in EQBO winters, which is often measured by the ratio of rain rate to OLR anomalies^39, 42^. The difference in cloud optical depth is not as robust as in CTP or atmospheric radiation budget (Fig. S9), suggesting that the difference in atmospheric radiation budget can largely be attributed to the rise of cloud top heights.

In the WP region, the grid-mean precipitations for regime 1 grid cells are higher in EQBO winters (Fig. S7i; *p*-value = 0.014). Also, while the results are broadly consistent with those in the MC region, the p-values of the energy budget terms associated with the differences between EQBO and WQBO winters are higher (i.e., lower confidence levels; Fig. S13). Combining these two results, EQBO-WQBO difference in longwave cloud-radiative feedback appears to be weaker in the WP region since the reduction in OLR is accompanied by an increase in precipitation.

### Why is the QBO-MJO connection exclusive to DJF, and stronger in the MC area?

Our results suggest that for QBO-induced lower-stratospheric temperature and static stability changes to significantly affect tropical deep convective systems associated with the MJO, the following two conditions must be satisfied: (i) temperature perturbations associated with QBO are strong enough to alter the properties of deep convection, and (ii) a sufficiently large number of convective towers that can potentially reach the altitude in which temperature is modulated by the QBO exist. It turns out that the above two conditions are satisfied most robustly during DJF and in the MC region.

**Fig. 8:**
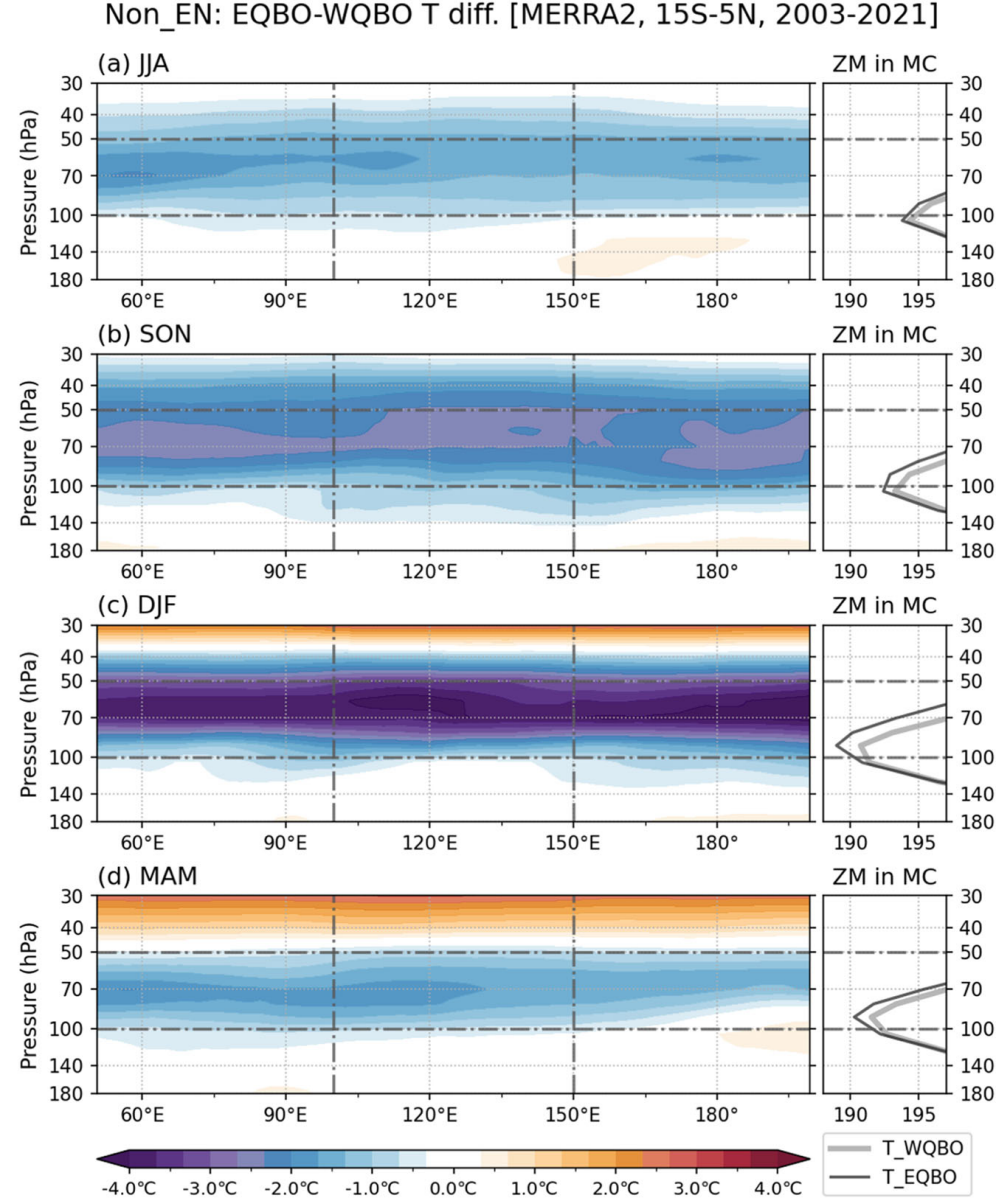
Seasonal variability of Quasi-Biennial Oscillation (QBO)-associated temperature anomalies in the upper troposphere and lower stratosphere. Meridional (15°S−5°N) mean upper temperature differences between the Non-El Niño easterly QBO (EQBO) and westerly QBO (WQBO) conditions for the four seasons: (**a**) June–August, (**b**) September–November, (**c**) December–February, and (**d**) March–May. 50 and 100 hPa levels are highlighted with horizontal dash lines, while vertical dashed lines indicate the longitudinal boundaries of the Maritime Continent (MC) domain (100°E-150°E). The smaller panels on the right side show zonal mean temperature profile in the MC domain, WQBO (gray) and EQBO (black).

Figure 8 shows that the QBO-associated temperature differences exhibit distinctive seasonality and regionality; the magnitude of temperature differences and associated changes in static stability is overall larger in DJF than in the other seasons (Fig. 8c). While studies have documented the distinct seasonal cycle of QBO temperature anomalies^52^, its origin remains elusive^53^. Martin et al.^53^ found that the seasonal cycle of QBO temperature anomalies cannot be explained by the seasonality in MJO activity, the phase locking between the QBO and the annual cycle, or the large-scale stratospheric circulation. Also, the UTLS temperature anomalies during EQBO years is notable only in the deep tropics regardless of the season^20,53^. Therefore, if QBO-associated temperature and static stability changes were to affect the vertical extent of deep convection, the effects should be the strongest in DJF and in deep tropics.

**Fig. 9:**
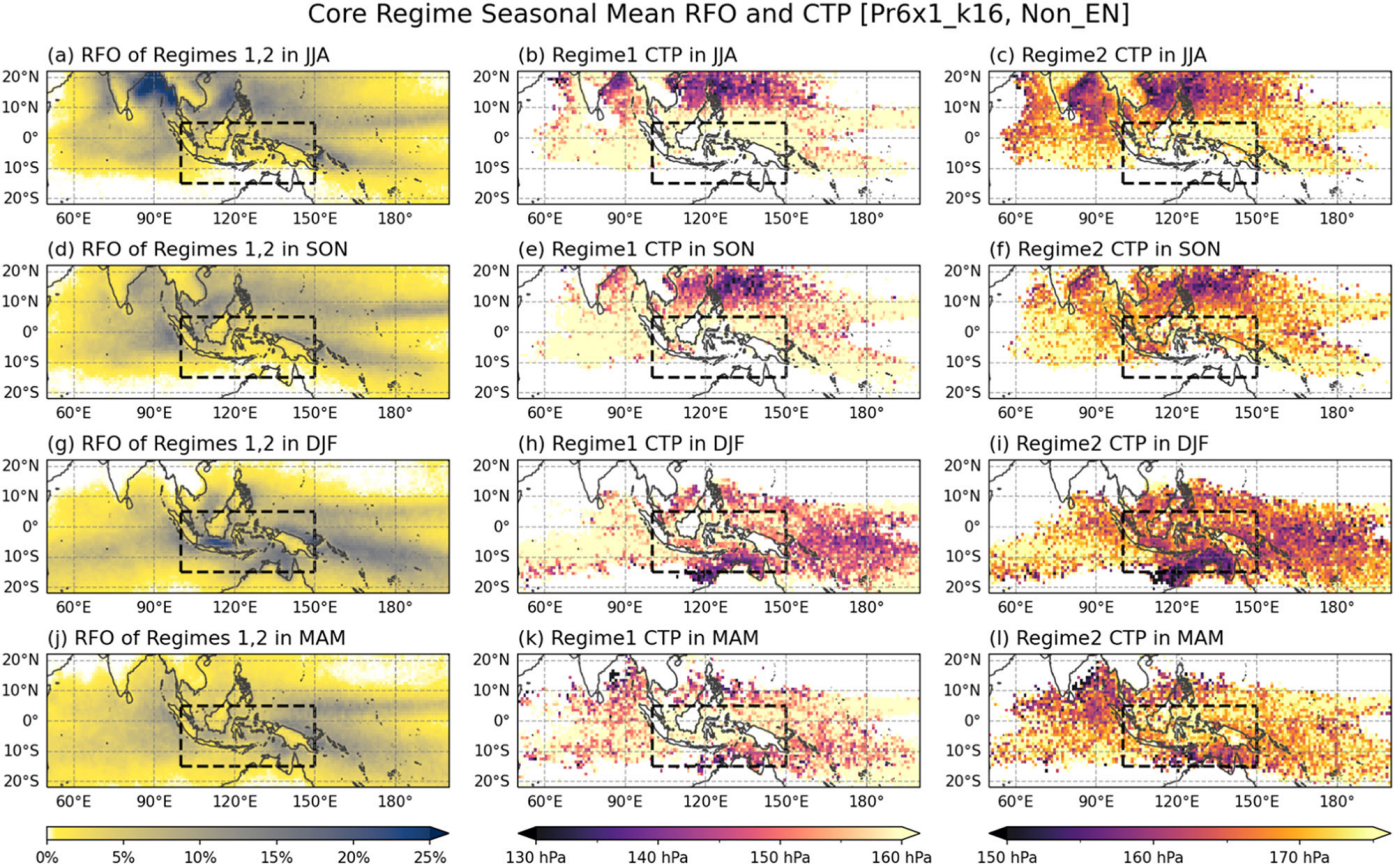
Seasonal variability of the population and height of deep convective systems. Seasonal climatology of Core regimes in the case of Non-El Niño condition: (**a**, **d**, **g**, **j**) combined relative frequency of occurrence (RFO) of regimes 1 and 2, (**b**, **e**, **h**, **k**) mean cloud top pressure (CTP) of regime 1, and (**c**, **f**, **i**, **l**) mean CTP of regime 2, for (first row) June–August, (second row) September–November, (third row) December–February, and (bottom row) March–May. The black dashed line box delineates the Maritime Continent domain (100°E−150°E, 15°S−5°N).

Moreover, the population of deep convective systems whose tops are in the upper troposphere varies strongly with the season. Figure 9 shows that the climatological seasonal cycle of RFOs of the Core regimes and the corresponding mean CTPs. The peaks in the occurrence frequency of the Core regimes feature meridional undulation following the seasonal march of the upward branch of the Hadley circulation. During DJF, the deep convective regimes are most populous in the deep tropical MC and WP regions (Fig. 9g), over which the warmest SST and coldest tropopause temperature are observed^54, 55^. In contrast, during JJA and SON, they are active mostly in the Indian and west Pacific monsoon regions to the north of 15^o^N, where intraseasonal variability has a strong northward propagating signal^56^. The mean CTP patterns of the Core regimes are generally consistent to the RFO patterns. In DJF, deep convective systems having lower CTP occur in the equatorial belt in which QBO temperature anomalies are most pronounced, while they move to north in JJA and SON. During DJF, the mean CTP of deep convective systems is notably higher (i.e., lower altitude of cloud top meaning weaker convection) in the IO region than in the MC and WP regions, which may explain why the EQBO-WQBO contrast in cloud properties is particularly insignificant there.

The results of Figs. 8 and 9 may suggest that a statistically robust QBO-MJO connection appears almost exclusively during DJF because it is the season when EQBO temperature anomalies are the strongest and deep convective systems are most frequent in the deep tropical MC and WP regions, with the deepest systems occurring more frequently in the MC region. Thus, for climate models to correctly simulate the QBO-MJO connection, they must be able to represent the seasonal cycle of the population of deep convective cloud systems as well as the seasonality of the QBO temperature anomalies.

## Discussion

The current study provides robust observational evidence of the QBO-induced MJO convection changes, which support the hypothesis that the enhancement of MJO convective activity in EQBO winters is due to the QBO-induced thermal structure changes in the UTLS. The QBO-induced temperature anomalies during EQBO winters deepen further deep convective systems, allowing them to strengthen and become more populous through the longwave cloud-radiation feedback, which enhances the propagation of the MJO across the MC region. Our results also offer useful insights into the seasonality and regionality in the QBO-MJO relationship. We find that the magnitude of QBO-induced temperature anomalies and the frequency of deep convective systems that reach the UTLS region were the highest in the MC region during DJF, in which the QBO-MJO connection is most pronounced. The QBO-induced enhancement of MJO convection in the MC region may allow the MJO to overcome the ‘MC blocking effect’ on the eastward propagation of the MJO^57–59^.

Consistent with several recent studies, our results suggest that QBO modulates MJO by altering the intensity of LW cloud-radiation feedback. Sakaeda et al.^35^ previously hypothesized that MJO was most strongly affected by QBO among the family of the convectively coupled waves in the tropics due to the critical role of LW cloud-radiation feedback as a maintenance mechanism. It is worthwhile noting, however, that some aspects of the effects of enhanced LW cloud-radiation feedback during EQBO winters are not fully understood. In their idealized model simulations, Nie and Sobel^48^ found an increase of upper-tropospheric convective mass flux and cloud fraction in response to the reduced static stability in the UTLS. They also showed that the vertical profile of the large-scale vertical motion became more top-heavy, which favors an increase in the effective gross moist stability, and thereby weakens the large-scale waves associated with the vertical motion such as MJO. We speculate that this weakening effect of enhanced LW cloud-radiation feedback may be smaller than its corresponding strengthening effect for the MJO, which has the most top-heavy vertical velocity structure among the known convectively coupled tropical waves^35,60^. In any case, further work is needed to elucidate the role of changes in the shape of vertical velocity anomalies due to enhanced LW cloud-radiation feedback. Also, as different views on the nature of the MJO exist^15,16^, more work needs to be done to explore other processes that can potentially influence the QBO-MJO coupling.

We argue that the enhanced LW cloud-radiation feedback during EQBO winters (Figs. 5 and 7) is mainly due to further deepening of deep convective systems, which would increase the amount of ice cloud at higher altitudes. While our results are consistent with those from numerical experiments^36,48^, we cannot rule out the possibility that other mechanisms are involved. For example, Lin and Emanuel^61^ suggested that the modulation of ice clouds in the upper troposphere by MJO-induced Kelvin and Rossby waves is crucial for the LW cloud-radiation feedback of the MJO. In their simple model of the troposphere-stratosphere coupled system, QBO-associated wind anomalies alter the magnitude of MJO-induced waves and associated ice cloud anomalies in the upper troposphere, thereby affecting the model’s growth rate of the MJO-like mode. Our results as well as those of Lin and Emanuel^61^ warrant further investigations of how the QBO affects the MJO modulation of ice clouds in the UTLS, with a focus on the relative role of vertical transport of ice hydrometeors versus wave-induced cooling.

A few caveats of our study and ideas for potential future studies are discussed here. Our study focuses exclusively on a specific mean state change, namely QBO-induced temperature change in the UTLS. Therefore, it would be appropriate to examine other mean state variables that are known to have strong influence on MJO activity over the MC region. A preliminary analysis reveals no meaningful difference in the sign and magnitude of the seasonal mean anomalies of sea surface temperature and precipitable water (Figs. S17 and S18). This result indicates that sea surface temperature and precipitable water mean state changes during QBO winters do not explain the systematic difference in cloud properties between EQBO and WQBO winters.

We excluded the winters with a notable El Niño signal from our analysis because the contrast between EQBO and WQBO in UTLS temperature changes weakens as the El Niño signal strengthens (Fig. 1c). While removing strong El Niño winters allowed for a cleaner comparison between EQBO and WQBO winters, it also reduces the sample size. Although our main results (Figs. 5 and 7) pass the statistical significance test despite relatively small sample sizes (59 days for WQBO winters and 44 days for EQBO winters), a re-examination of our results with a longer observation record is warranted. The nonlinear influence of ENSO on UTLS temperature in the MC region also raises important questions, such as why T50-100 differences between EQBO and WQBO winters decrease when El Niño is strong. Considering the known influence of ENSO on lower-tropospheric temperature via the Brewer-Dobson circulation^62–64^, more work is needed to better understand how El Niño interferes with QBO signal in the UTLS of the deep tropics.

Investigating the entire lifecycle of the deep convective systems may provide useful insights into how they grow deeper under the EQBO state, which is not possible with observations from polar-orbiting satellites. It will be helpful particularly to address the question of why the deep convective systems in the MC region develop deeper than in the other regions during DJF (Fig. 9h, i). While covering the entire globe with higher resolution than their geostationary counterparts, polar-orbiting satellites are limited to one pass per each illuminated part of the day at low latitudes. Expanding our analysis into observations from geostationary satellites (e.g., Himawari-8) will offer an opportunity to confirm our results with more diurnally complete observations.

Realistic representation of the observed QBO-MJO connection is a daunting task for most, if not all, contemporary GCMs^33^. Our observational results can aid model development toward improving the simulation of the QBO-MJO connection, by providing a reference against which models can be evaluated and tuned. Improving the QBO-MJO connection in global models will eventually improve the prediction of MJO-related high-impact weather and climate events.

## Methods

### Satellite observations and reanalysis data

Cloud properties are retrieved from measurements by the Moderate Resolution Imaging Spectroradiometer (MODIS) instrument aboard the Terra and Aqua satellites. The MODIS cloud product (MOD08_D3 and MYD08_D3)^65–67^ provides Level-3 cloud observations at daily time scales with 1° × 1° horizontal resolution. We specifically use grid-mean daytime cloud top pressure (CTP), cloud optical thickness (COT), and the 2D joint histogram of COT and CTP. The joint histogram is composed of cloud fraction (CF) values for a total of 42 histogram bins (combining 6 COT and 7 CTP classes). We use the recent version Collection 6.1^67,68^. The 2D joint histograms used in this study include the sum of the Partially Cloudy (PCL) and nominal joint histograms, as in previous studies^69,70^.

Rain rate is obtained from the Integrated MultisatEllite Retrievals for GPM (IMERG) data, which provides seamless precipitation estimates at a 0.1° grid every half hour by unifying observations from a network of partner satellites in the GPM constellation^71^. The most recent major version, V06B^72^, covers an extended period from June 2000 onward by including TRMM data in the pre-GPM era. In this study, we use the Final run product which is of the best quality among IMERG products by virtue of utilizing all relevant observations including monthly gauge data. It is worthwhile to note that, since this study mostly focuses on clouds that produce relatively heavy rainfall, the effect of the known tendency of IMERG to overestimate light precipitation^73,74^ is minimal.

Radiative fluxes are obtained from Clouds and the Earth’s Radiant Energy System (CERES) synoptic top-of-atmosphere (TOA) and surface fluxes and clouds (SYN) 1-deg product^75^ while brightness temperature (BT) comes from NCEP/CPC Merged IR dataset^76^. Atmospheric temperature and wind come from MERRA-2 reanalysis^77^. The neutral buoyancy level (NBL) is calculated using the function EL() in MetPy^78,79^. We use total 34 levels (up to 30hPa) of air temperature and relative humidity from MERRA-2 Tavg3_3d_asm_Nv dataset, after transformed from model levels to pressure levels. The bottom level values (at 1000hPa) are replaced by surface pressure, 2 m air temperature and relative humidity before applying the EL function. For the T Modification experiment, mean T difference in the MC domain is prepared using WQBO and EQBO seasonal means (Non-El Niño winters; Fig. 2). For the WQBO case, the mean T difference, EQBO-WQBO is added to individual T profiles at lower than 100hPa pressure level, while it is subtracted for the EQBO case.

Whenever necessary, all data are spatio-temporally matched to Terra or Aqua satellite passes unless noted otherwise. We focus on DJF for the period of December 1980 to February 2021 (41 seasons). For the satellite data analyses, the period had to be shortened to 19 seasons starting from December 2002.

### Cloud-precipitation regime

Our analysis of cloud properties is based on the cloud-precipitation regimes (CPRs). The CPRs are a convenient means to characterize the combined cloud and precipitation formations within a 1-degree grid cell. These CPRs were developed recently by Jin et al.^70^ (J21 hereinafter) by combining MODIS 2D joint histograms of COT and CTP with spatio-temporally matched precipitation histograms from the IMERG precipitation product in the regime classification. In this study, we use the (Cld42 + )Pr6x1 set in J21, which is the set of natural concatenation of cloud (42 bins) and precipitation (6 bins) histograms that gives a 7-to-1 ratio of cloud and precipitation weights. J21 showed that, compared to the cloud-only regime, the cloud-precipitation regimes provide more precise grouping with the added precipitation information while maintaining distinct cloud histogram patterns. Note that, when constructing the regime dataset through *k*-means clustering^80,81^, we use both Terra and Aqua observations available on each day and grid cell, while we average the two (when both are available) for the grid cell assignment process. This ensures that the fraction of missing data in tropical warm pool domain is minimal (~1.5%). All other variables matched to Terra/Aqua passes are also averaged in the same manner, and 24-h mean is defined as the average in the period of ±12 h centered on local noon.

Figure S19a–d shows Pr6x1 centroids for selected CPRs. From a total of 16 regimes in the Pr6x1 set shown in J21, we use the regimes that are associated with tropical deep convection, and closely related to MJO (regimes 1, 2, 4, and 6; see Fig. S3). The selected four regimes have commonly the lowest values of CTP (highest altitude) and represent nearly overcast conditions, namely grid-cell total cloud fraction above 95% except regime 6 which is closer to 90%. Regime 1 is composed of the optically thickest clouds representing full of convective core in a 1-deg grid-cell, and regime 2 is composed of the second thickest clouds representing the mixture of convective core and nearby thick stratiform clouds. These two regimes are referred to as Core regimes. Regimes 4 and 6 have lower COT for similar CTPs and are referred to as Anvil regimes.

Each regime’s relative frequency of occurrence (RFO) map is shown in Fig. S19e–h. These RFO maps are somewhat different from the all-season RFO maps shown in J21 because they are calculated only for boreal winter. The RFO maps show clearly large-scale ascending branches in boreal winter: the Intertropical Convergence Zone (ITCZ), South Pacific Convergence Zone (SPCZ), and Indo-Pacific warm pool regions, as well as continental signals in Amazon basin and equatorial Africa. A key difference between regime 1 and other regimes is that regime 1 occurs rarely in the Maritime Continent. Two potential reasons explain this scarcity: first, deep convection that is horizontally aggregated in large areas so as to fill a 1-deg grid cell occurs more often over open ocean rather than land^82^; and second, deep convection and associated heavy rainfall activity are relatively weaker around the time of Terra and Aqua overpass (near local noon) in the Maritime Continent^83^.

### Indices for MJO, QBO, and ENSO

We employ the Real-time Multivariate MJO (RMM) index of Wheeler and Hendon^84^ for MJO identification. On a given day, the RMM index consists of two scalar numbers with which the intensity (amplitude) and geographical location (phase) of MJO convective envelope can be identified. Our analysis focused on when anomalous convection associated with the MJO is in the Maritime Continent (MJO phase 4 or 5) as it has been shown that the QBO modulation of MJO activity is the strongest in that region. As in many previous studies, we use seasonally and meridionally averaged in 5°S-5°N, zonal mean zonal wind at 50 hPa (U50) to categorize each DJF season into EQBO (U50 < −7 ms^−1^), WQBO (U50 > 7 ms^−1^), and QBO-neutral (other values). We also use seasonally averaged Niño 3.4 index to characterize the state of El Niño/Southern Oscillation (El Niño, La Niña, ENSO-neutral) in each boreal winter season. We excluded winters with averaged Niño 3.4 index >0.5 (referred to as Non-El Niño; see Table 1).

## Supplementary information

supplementary information

## Data Availability

The cloud-precipitation regime dataset (including centroids, CPR-number-on-map, and UTC info) is available at https://data.nasa.gov/dataset/Tropical-Cloud-Precip-hybrid-Regime-Pr6x1-set/h5pe-bcfp. IMERG precipitation data (V06 Final Run; 10.5067/GPM/IMERG/3B-HH/06), NCEP/CPC Merged IR data (10.5067/P4HZB9N27EKU), and T and U profile data of MERRA-2 (tavg3_3d_asm_Nv, 10.5067/SUOQESM06LPK) were obtained from Goddard Earth Sciences Data and Information Services Center (GES DISC; https://disc.gsfc.nasa.gov/), Greenbelt, MD, USA. The Level-3 (L3) MODIS Atmosphere Daily Global Product of Terra (MOD08_D3, 10.5067/MODIS/MOD08_D3.061) and Aqua (MYD08_D3, 10.5067/MODIS/MYD08_D3.061) was obtained from the Level-1 and Atmosphere Archive & Distribution System (LAADS) Distributed Active Archive Center (DAAC; https://ladsweb.modaps.eosdis.nasa.gov/) in the Goddard Space Flight Center, Greenbelt, MD, USA. CERES SYN1deg - Level 3 data (10.5067/TERRA+AQUA/CERES/SYN1DEG-1HOUR_L3.004A) was obtained from https://ceres.larc.nasa.gov/data/. The Real-time Multivariate MJO (RMM) index data is available at http://www.bom.gov.au/climate/mjo/graphics/rmm.74toRealtime.txt, and Niño3.4 SST index is provided by the NOAA/OAR/ESRL PSL, Boulder, Colorado, USA, from their Web site at https://psl.noaa.gov/gcos_wgsp/Timeseries/Nino34/. Source data are provided with this paper.

## Source data

Source Data

## Code availability

Python (version 3.9) was used to produce all figures. The neutral buoyancy level (NBL) was calculated using MetPy (version 1.4), an open-source Python package available at https://unidata.github.io/MetPy/latest/index.html.
