# Supplementary material for: QBO deepens MJO convection
Source: Nat Commun. 2023 Jul 10;14:4088. doi: 10.1038/s41467-023-39465-7 (PMC10333186; doi:10.1038/s41467-023-39465-7)
Supplement: Supplementary file 1 — supplementary information [file 41467_2023_39465_MOESM1_ESM.pdf]

[Supplementary information for]
QBO deepens MJO convection

Daeho Jin[1,2], Daehyun Kim[3*], Seok-Woo Son[4], Lazaros Oreopoulos[2]

[1] University of Maryland - Baltimore County, Baltimore, MD, USA

[2] Earth Sciences Division, NASA's Goddard Space Flight Center, Greenbelt, MD, USA

[3] University of Washington, Seattle, WA, USA

[4] Seoul National University, Seoul, South Korea

*Corresponding author: Daehyun Kim (daehyun@uw.edu)

Contents of this file

Figures S1 to S19

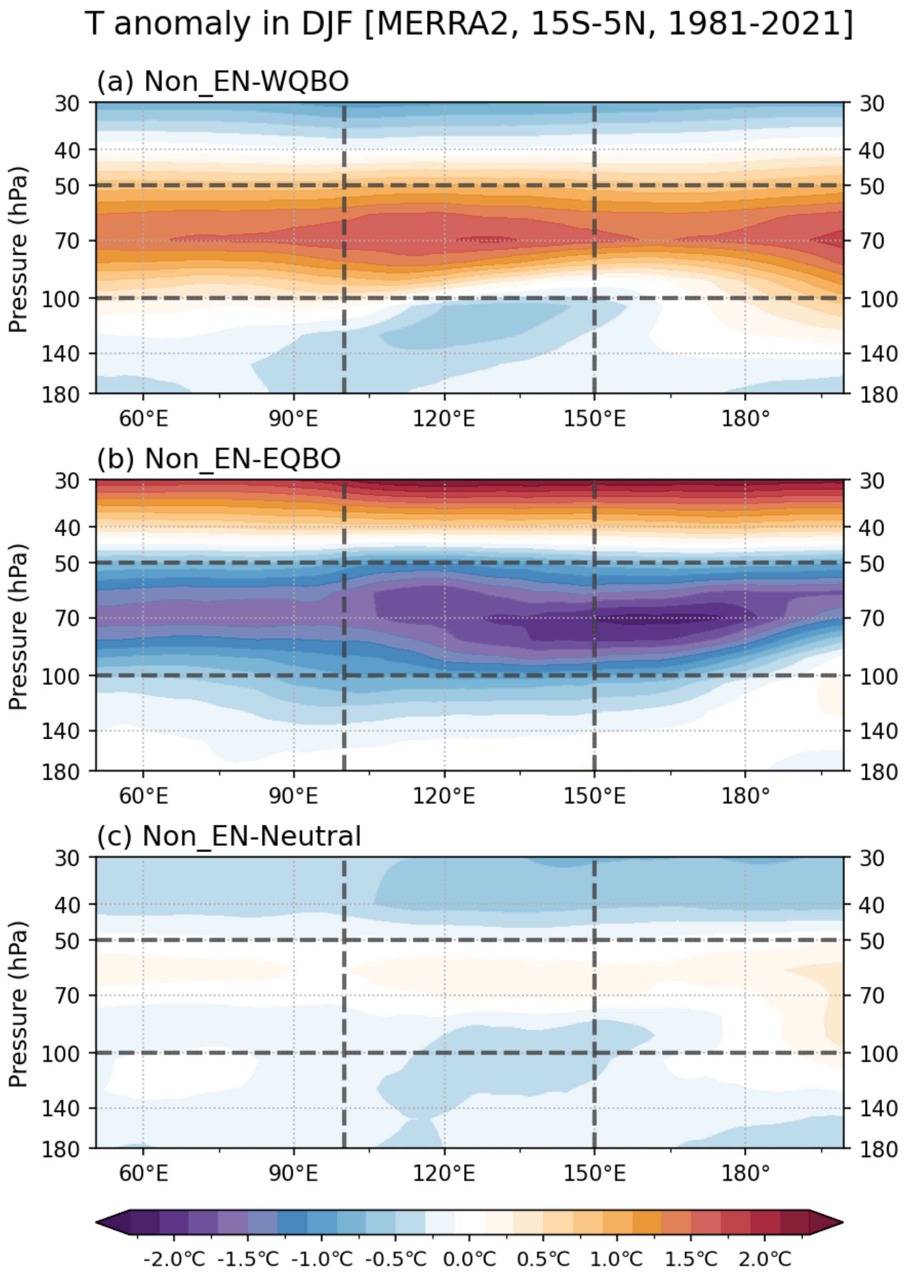

Figure S1. Temperature anomalies associated with Quasi-Biennial Oscillation (QBO) during boreal winter in the upper troposphere and lower stratosphere. December to February (DJF) meridional (15°S–5°N) mean anomalies of upper atmosphere temperature composited for combined conditions of Non-El Niño and (a) westerly QBO (WQBO), (b) easterly QBO (EQBO), and (c) QBO-neutral. The 50 and 100 hPa levels are highlighted by horizontal dash lines, while vertical dashed lines delineate the longitudinal boundary of the Maritime Continent domain (100°E-150°E).

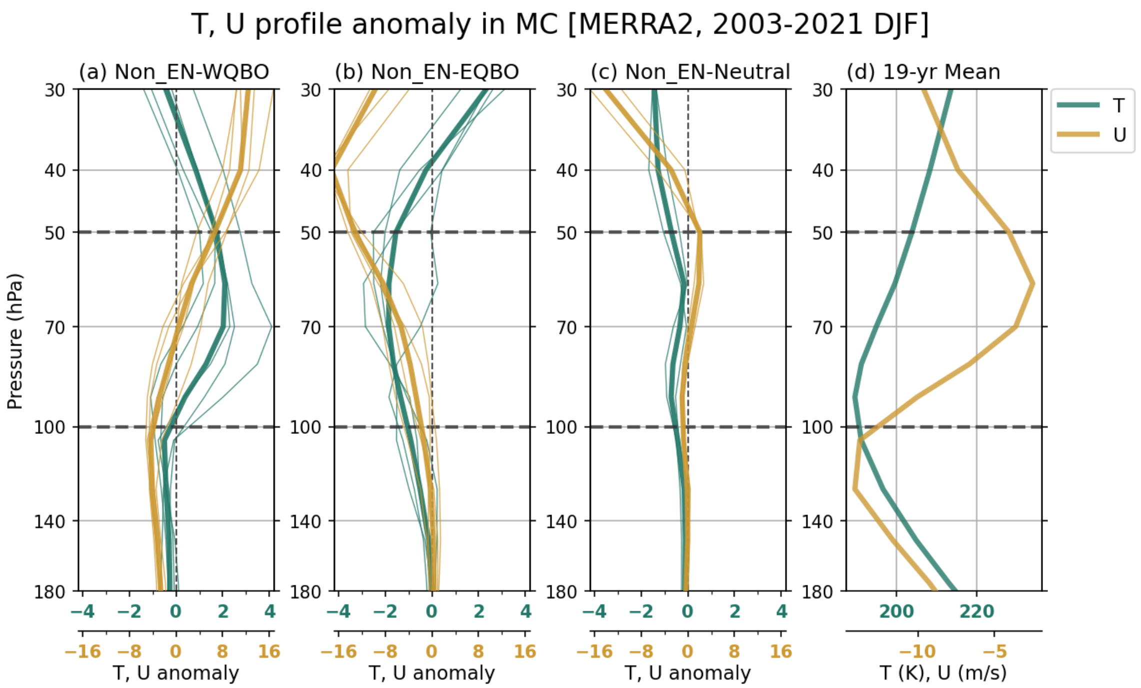

Figure S2. Quasi-Biennial Oscillation (QBO)-associated temperature and wind perturbations in the upper troposphere and lower stratosphere. December to February (DJF) mean seasonal anomalies of air temperature (T; green) and zonal wind (U; brown) averaged over Maritime Continent (MC; 100°E–150°E, 15°S–5°N) composited for (a) westerly Quasi-Biennial Oscillation (WQBO), (b) easterly QBO (EQBO), and (c) QBO-neutral states during Non-El Niño winters. Panel (d) shows the 41-year climatological DJF mean of T and U profiles (1981 to 2021). The think lines indicate the averages of the selected years (listed in Table 1). Note that this figure is same as Figure 2, but for a shorter period (2003-2021).

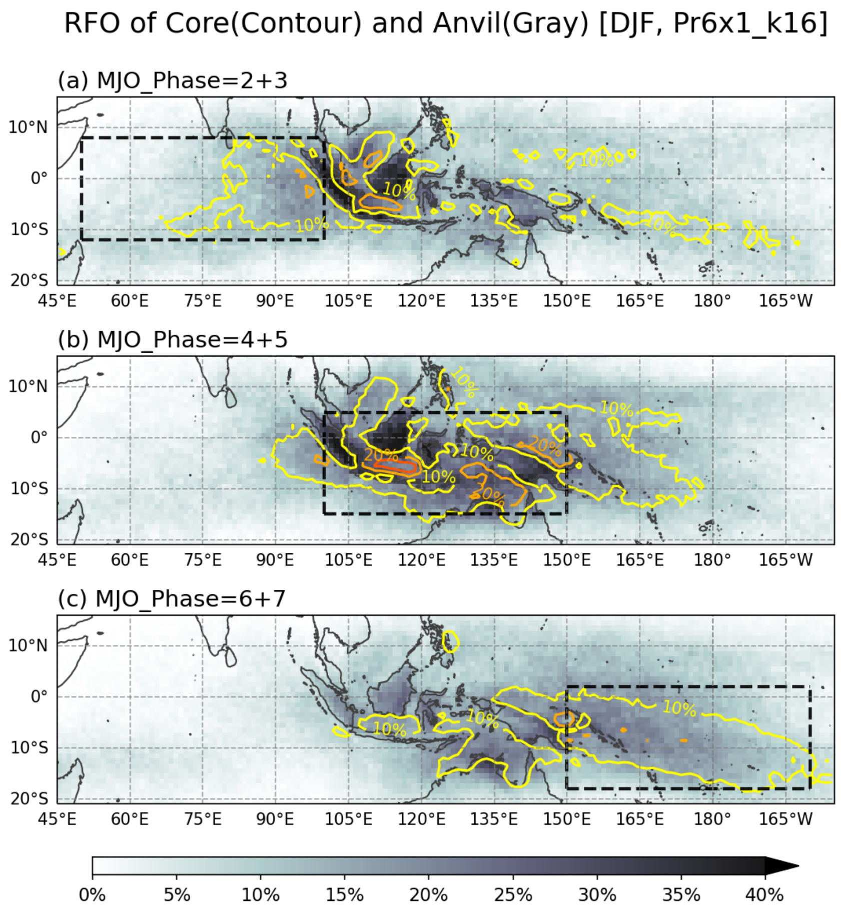

Figure S3. Modulation of the frequency of deep convective systems over the Indo-Pacific warm pool by the Madden-Julian Oscillation (MJO). Seasonal (December to February) relative frequency of occurrence (RFO) composite of convective core regimes (regime 1 and 2; contour) and anvil regimes (regime 4 and 6; gray shading) for (a) MJO phases 2 and 3, (b) MJO phases 4 and 5, and (c) MJO phases 6 and 7. The boxes in each panel outline the boundaries of the tropical Indian Ocean (TIO; 50°E–100°E, 12°S–8°N), MC (100°E–150°E, 15°S–5°N), and southwestern tropical Pacific (SWTP; 150°E–160°W, 18°S–2°N) domains.

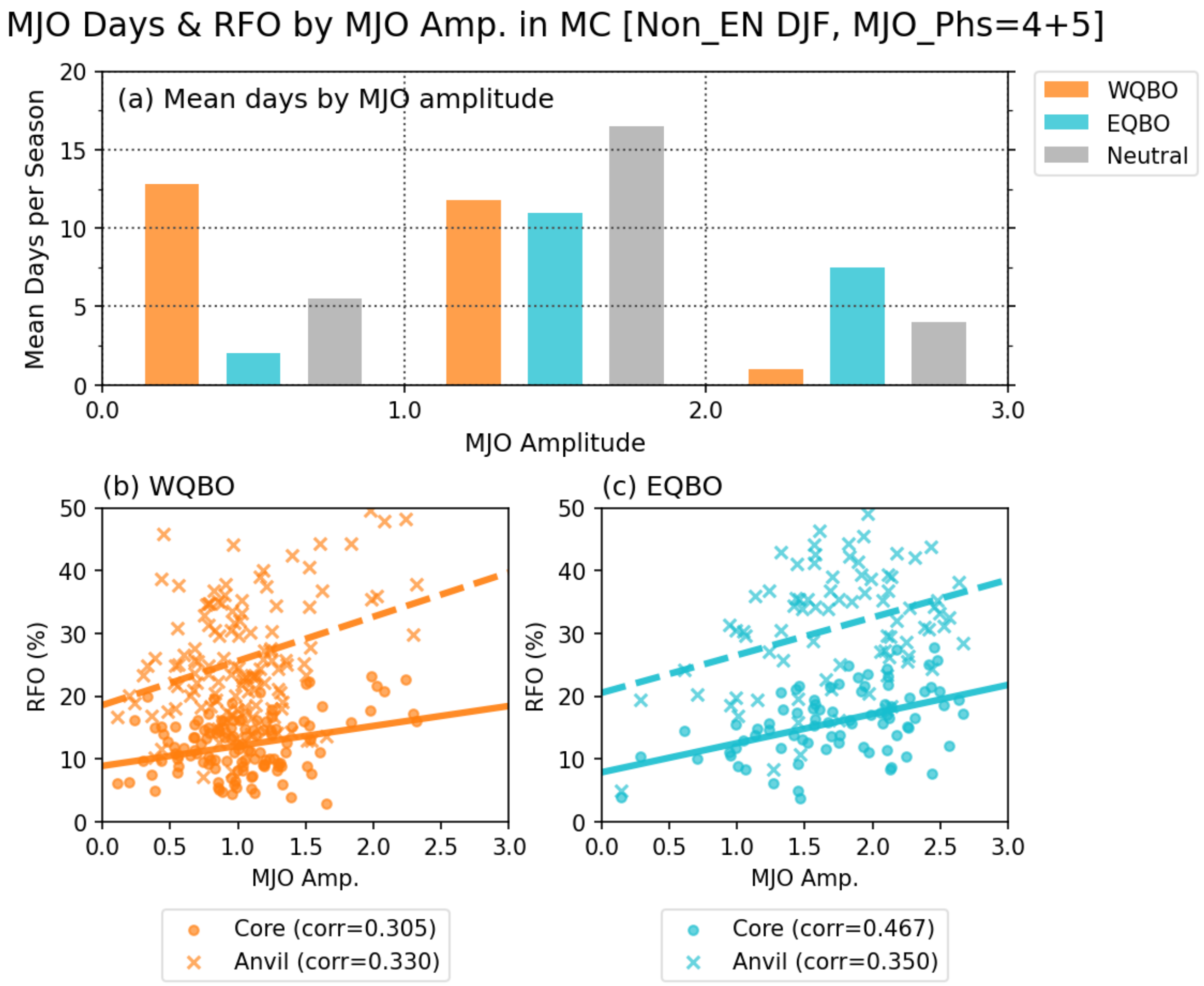

Figure S4. Relationship between Madden-Julian Oscillation (MJO) amplitude and the frequency of deep convective systems in the Maritime Continent (MC). (a) Mean days per December to February season satisfying combined conditions of specific MJO amplitude range, Non-El Niño and Quasi-Biennial Oscillation (QBO) conditions. (b)-(c) Scatter plots of (b) “Core (circle)” and (c) “Anvil (‘x’ symbol)” regimes mean relative frequency of occurrence (RFO) vs MJO amplitude in the MC (100°E–150°E, 15°S–5°N) domain. Days with MJO phases 4 or 5 are used.

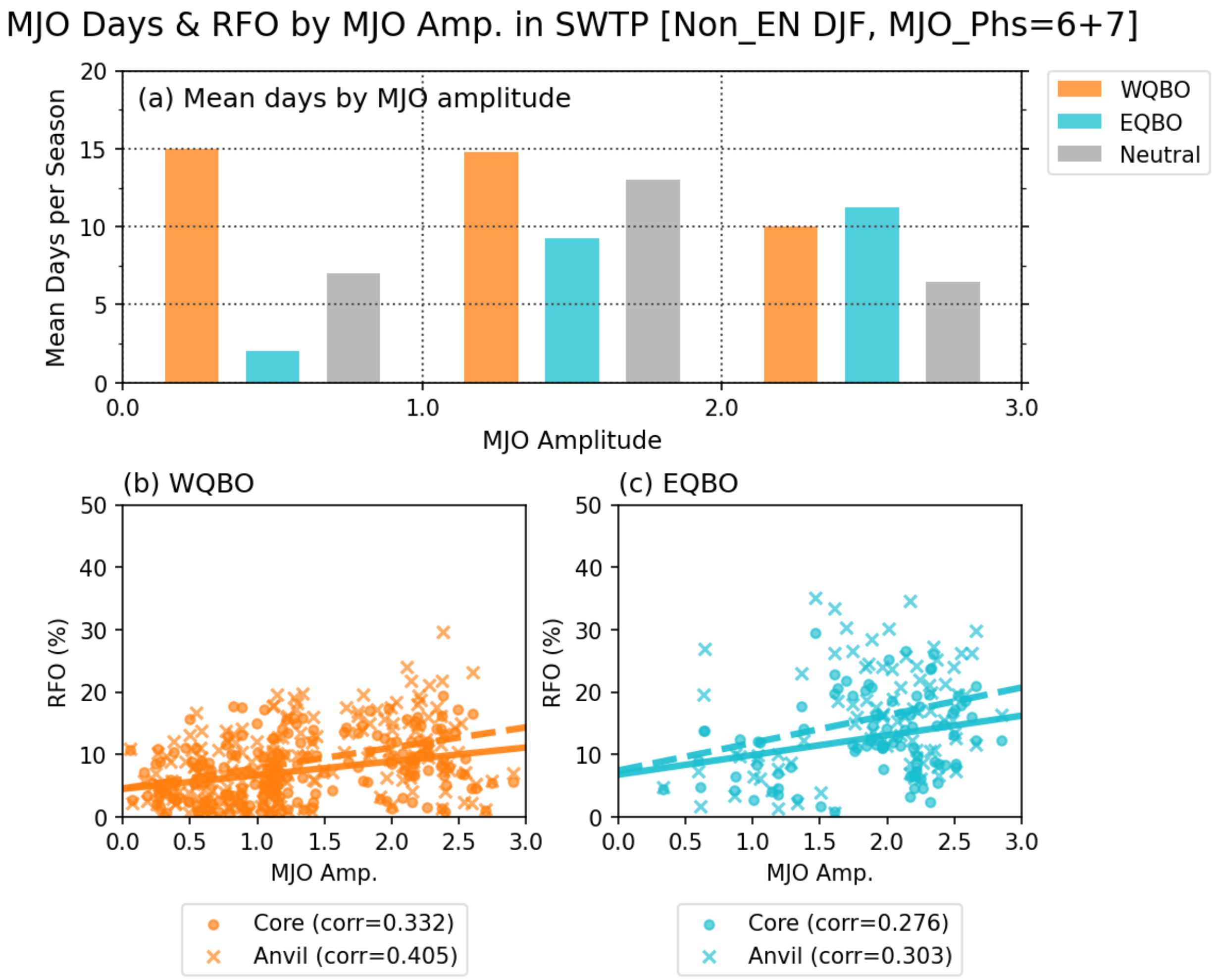

Figure S5. Relationship between Madden-Julian Oscillation (MJO) amplitude and the frequency of deep convective systems in the southwestern tropical Pacific (SWTP). (a) Mean days per December to February season satisfying combined conditions of specific MJO amplitude range, Non-El Niño and Quasi-Biennial Oscillation (QBO) conditions. (b)-(c) Scatter plots of (b) “Core (circle)” and (c) “Anvil (‘x’ symbol)” regimes mean relative frequency of occurrence (RFO) vs MJO amplitude in the SWTP (150°E–160°W, 18°S–2°N) domain. Days with MJO phases 6 or 7 are used.

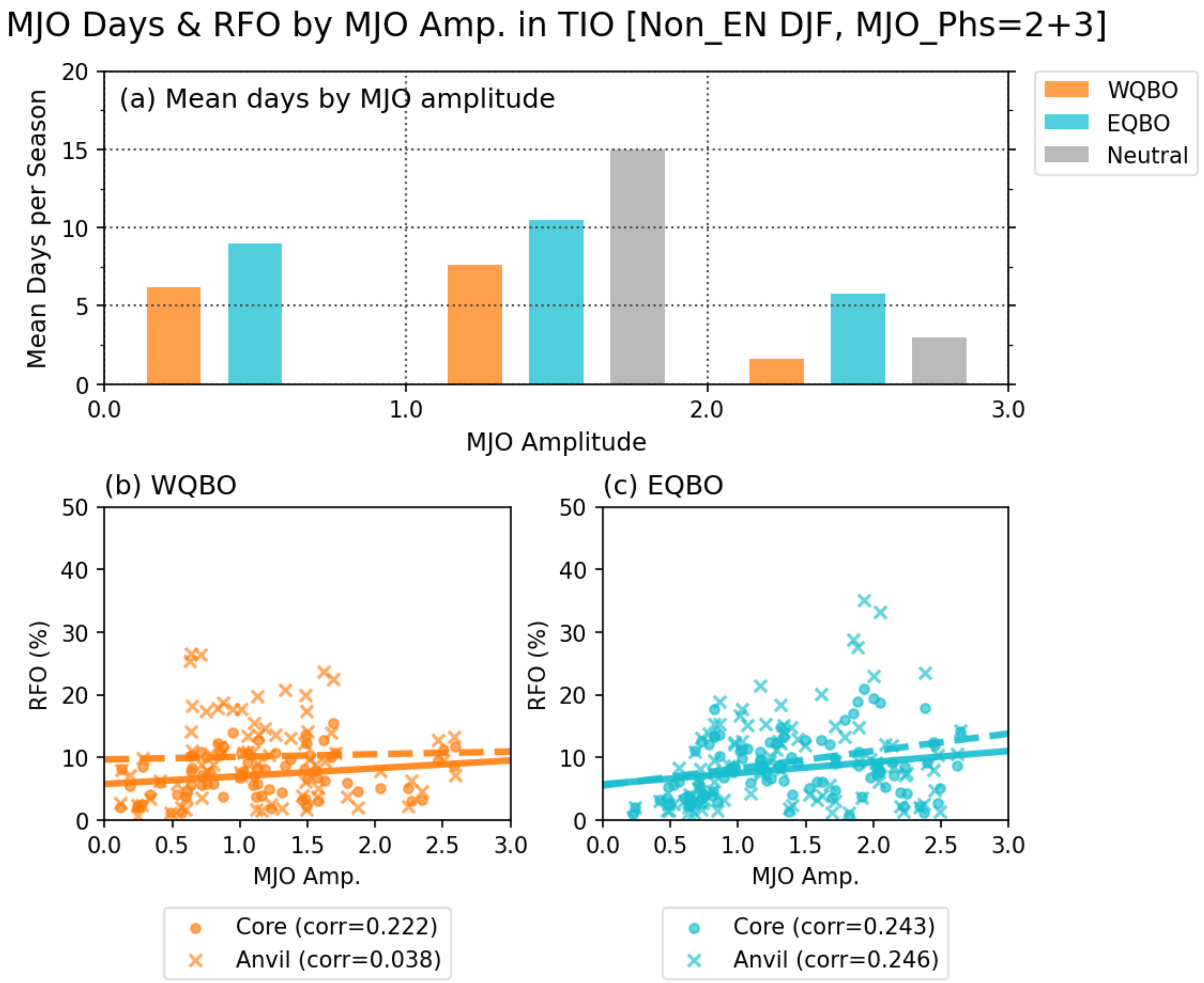

Figure S6. Relationship between Madden-Julian Oscillation (MJO) amplitude and the frequency of deep convective systems in the tropical Indian Ocean (TIO). (a) Mean days per December to February season satisfying combined conditions of specific MJO amplitude range, Non-El Niño and Quasi-Biennial Oscillation (QBO) conditions. (b)-(c) Scatter plots of (b) “Core (circle)” and (c) “Anvil (‘x’ symbol)” regimes mean relative frequency of occurrence (RFO) vs MJO amplitude in the TIO (50°E–100°E, 12°S–8°N) domain. Days with MJO phases 2 or 3 are used.

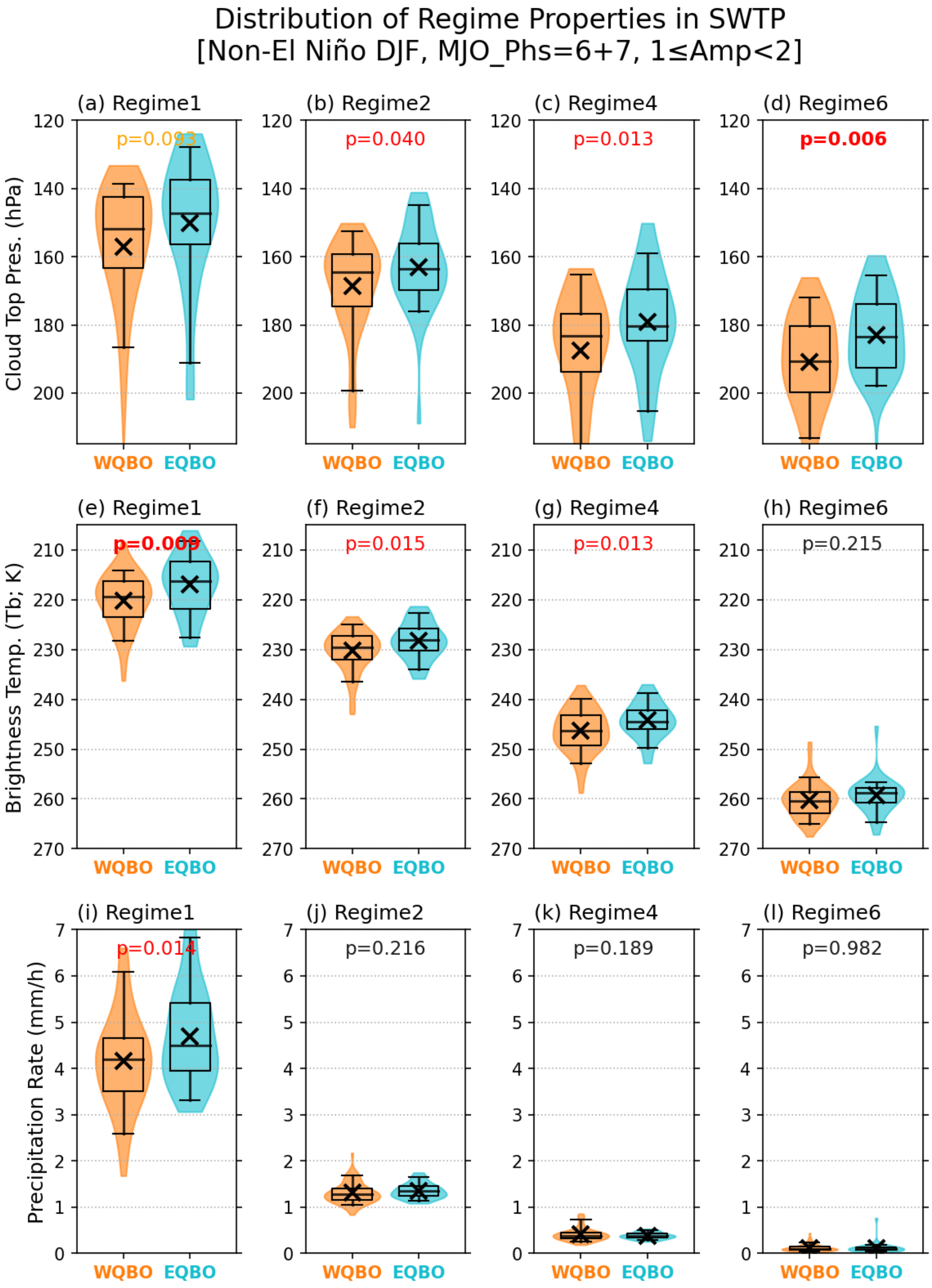

Figure S7. Comparison of cloud top pressure, brightness temperature, and precipitation in deep convective systems within Madden-Julian oscillation (MJO) envelops between easterly Quasi-Biennial Oscillation (EQBO) and westerly QBO (WQBO) winters. Distributions of southwestern tropical Pacific (SWTP; 150°E–160°W, 18°S–2°N) domain mean cloud top pressure (top row) and brightness temperature (middle row), and precipitation rate (bottom row) of grid cells identified as regimes (a), (e), (i) regime 1, (b), (f), (j) regime 2, (c), (f), (k) regime 4, and (d), (h), (l) regime 6, for WQBO (orange) and EQBO (blue) composite days, in violin-style box-whisker

plot (same convention as in Fig. 4). The conditions for compositing are simultaneous occurrence of MJO phases 6 or 7, and MJO amplitude in the 1-2 range during Non-El Niño winters. The significance of mean difference between WQBO and EQBO composites are obtained via a t-test, with the corresponding p-value shown at the top of each panel.

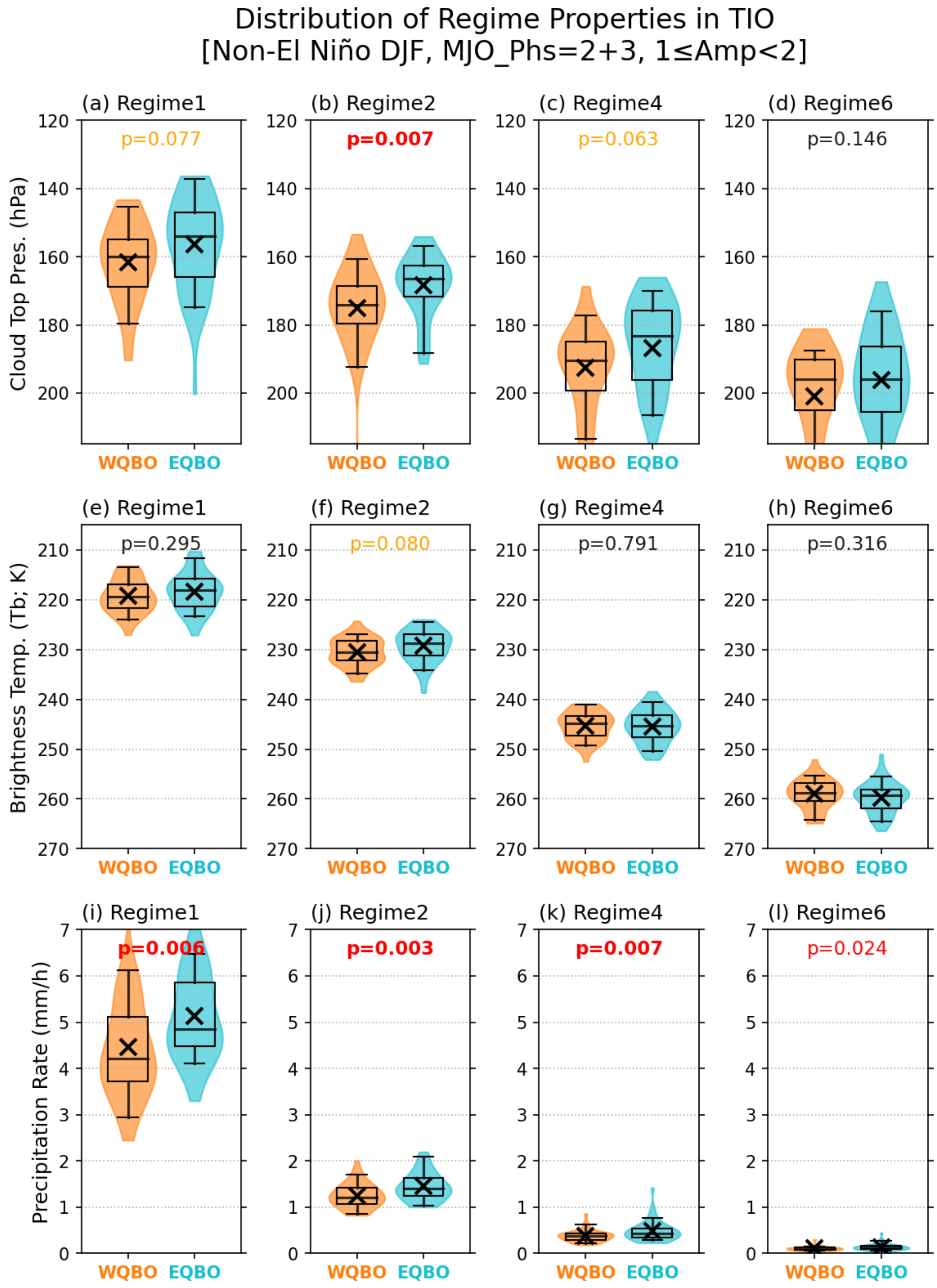

Figure S8. Comparison of cloud top pressure, brightness temperature, and precipitation in deep convective systems within Madden-Julian oscillation (MJO) envelops between easterly Quasi-Biennial Oscillation (EQBO) and westerly QBO (WQBO) winters. Distributions of tropical Indian Ocean (TIO; 50°E–100°E, 12°S–8°N) domain mean cloud top pressure (top row) and brightness temperature (middle row), and precipitation rate (bottom row) of grid cells identified as regimes (a), (e), (i) regime 1, (b), (f), (j) regime 2, (c), (f), (k) regime 4, and (d), (h), (l) regime 6, for WQBO (orange) and EQBO (blue) composite days, in violin-style box-whisker plot (same

convention as in Fig. 4). The conditions for compositing are simultaneous occurrence of MJO phases 2 or 3, and MJO amplitude in the 1-2 range during Non-El Niño winters. The significance of mean difference between WQBO and EQBO composites are obtained via a t-test, with the corresponding p-value shown at the top of each panel.

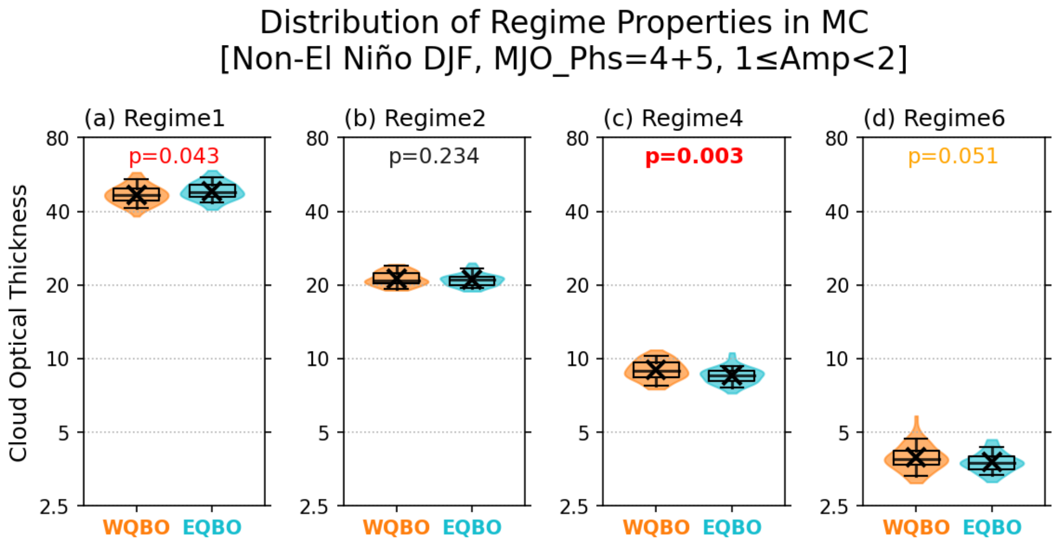

Figure S9. Comparison of cloud optical thickness (COT) in deep convective systems within Madden-Julian oscillation (MJO) envelops between easterly Quasi-Biennial Oscillation (EQBO) and westerly QBO (WQBO) winters. Distributions of Maritime Continent (100°E–150°E, 15°S–5°N) domain mean COT of grid cells identified as regimes (a) regime 1, (b) regime 2, (c) regime 4, and (d) regime 6, for westerly Quasi-Biennial Oscillation (WQBO; orange) and EQBO (blue) composite days, in violin-style box-whisker plot (same convention as in Fig. 4). The conditions for compositing are simultaneous occurrence of MJO phases 4 or 5, and MJO amplitude in the 1-2 range during Non-El Niño winters. The significance of mean difference between WQBO and EQBO composites are obtained via a t-test, with the corresponding p-value shown at the top of each panel.

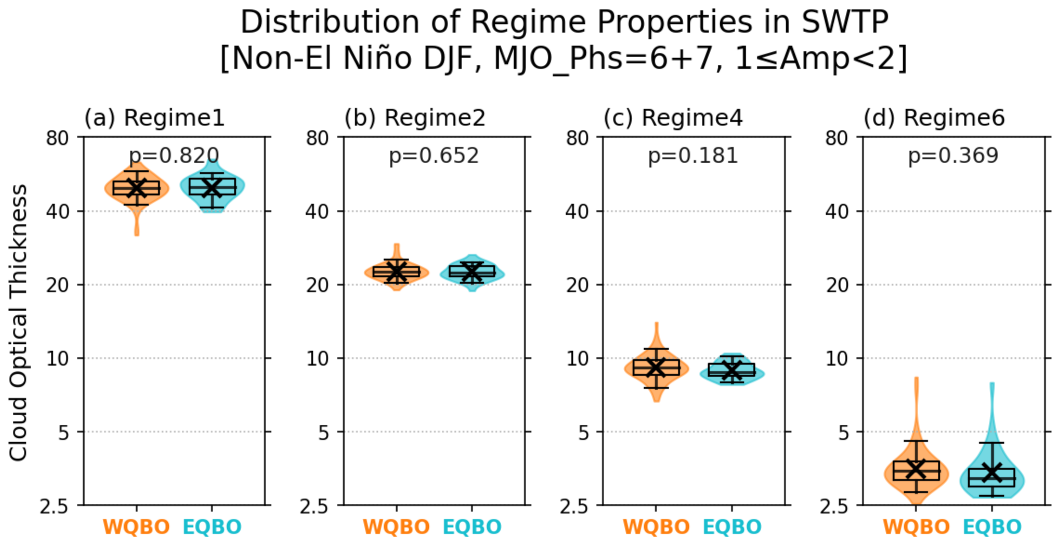

Figure S10. Comparison of cloud optical thickness (COT) in deep convective systems within Madden-Julian oscillation (MJO) envelops between easterly Quasi-Biennial Oscillation (EQBO) and westerly QBO (WQBO) winters. Distributions of southwestern tropical Pacific (SWTP; 150°E–160°W, 18°S–2°N) domain mean COT of grid cells identified as regimes (a) regime 1, (b) regime 2, (c) regime 4, and (d) regime 6, for westerly Quasi-Biennial Oscillation (WQBO; orange) and EQBO (blue) composite days, in violin-style box-whisker plot (same convention as in Fig. 4). The conditions for compositing are simultaneous occurrence of MJO phases 6 or 7, and MJO amplitude in the 1-2 range during Non-El Niño winters. The significance of mean difference between WQBO and EQBO composites are obtained via a t-test, with the corresponding p-value shown at the top of each panel.

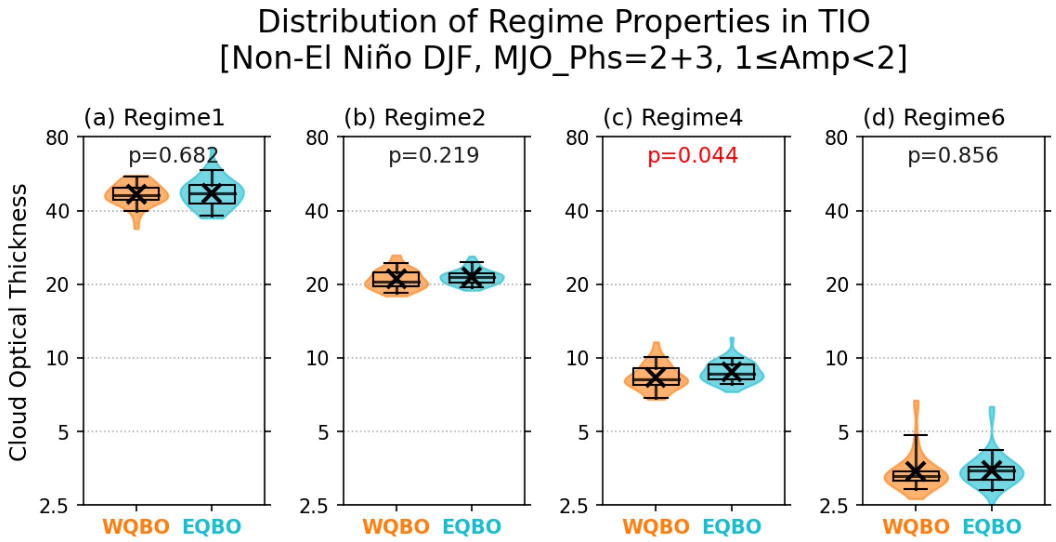

Figure S11. Comparison of cloud optical thickness (COT) in deep convective systems within Madden-Julian oscillation (MJO) envelops between easterly Quasi-Biennial Oscillation (EQBO) and westerly QBO (WQBO) winters. Distributions of tropical Indian Ocean (TIO; 50°E–100°E, 12°S–8°N) domain mean COT of grid cells identified as regimes (a) regime 1, (b) regime 2, (c) regime 4, and (d) regime 6, for westerly Quasi-Biennial Oscillation (WQBO; orange) and EQBO (blue) composite days, in violin-style box-whisker plot (same convention as in Fig. 4). The conditions for compositing are simultaneous occurrence of MJO phases 2 or 3, and MJO amplitude in the 1-2 range during Non-El Niño winters. The significance of mean difference between WQBO and EQBO composites are obtained via a t-test, with the corresponding p-value shown at the top of each panel.

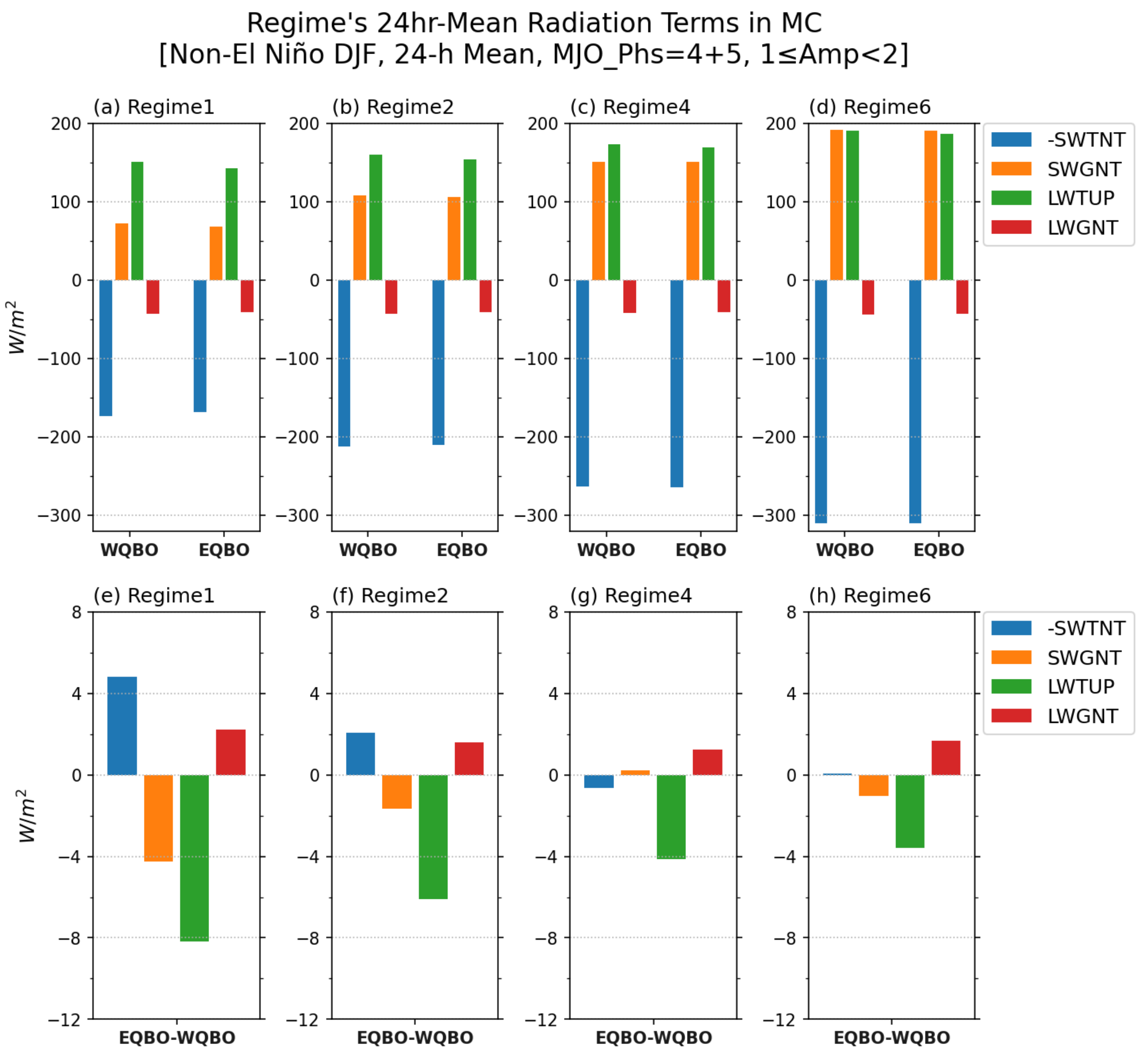

Figure S12. Comparison of radiative fluxes at the top of the atmosphere (TOA) and at the surface in deep convective systems within Madden-Julian oscillation (MJO) envelops between easterly Quasi-Biennial Oscillation (EQBO) and westerly QBO (WQBO) winters. (a)-(d) Distributions of Maritime Continent (100°E–150°E, 15°S–5°N) domain mean components of radiative flux divergence terms of grid cells identified as regimes (a) regime 1, (b) regime 2, (c) regime 4, and (d) regime 6, for WQBO and EQBO composite days. (e)-(h) Same as (a)-(d) but for the difference between WQBO and EQBO. The conditions for compositing are simultaneous occurrence of MJO phases 4 or 5, and MJO amplitude in the 1-2 range during Non-El Niño winters. "SWTNT" indicates net shortwave radiation at TOA, "SWGNT" net shortwave radiation at surface, "LWTUP" upward longwave radiation at TOA, and "LWGNT" net longwave radiation at surface. All terms are modified as positive meaning loss by atmospheric column.

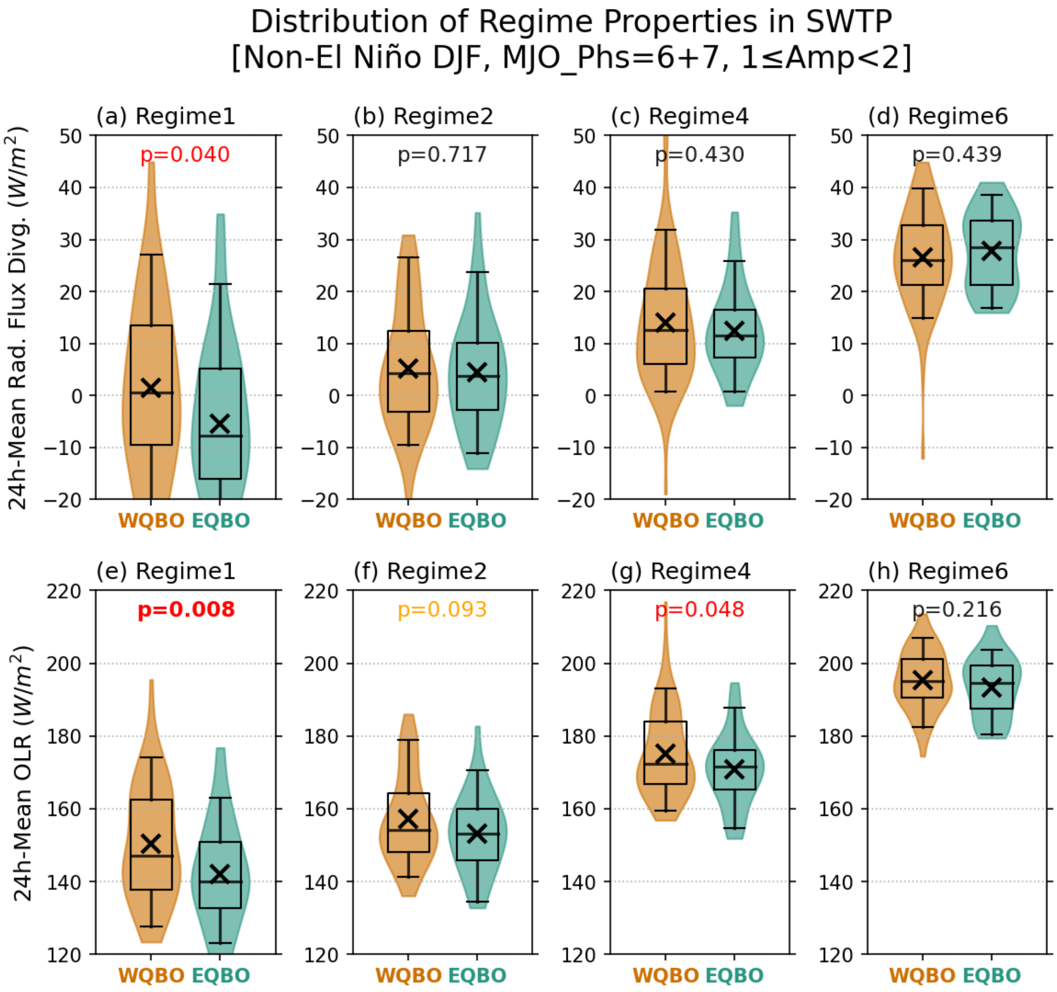

Figure S13. Comparison of column radiative flux convergence and outgoing longwave radiation (OLR) in deep convective systems within Madden-Julian oscillation (MJO) envelops between easterly Quasi-Biennial Oscillation (EQBO) and westerly QBO (WQBO) winters. Distributions of southwestern tropical Pacific (SWTP; 150°E–160°W, 18°S–2°N) domain mean cloud top pressure (top row) and brightness temperature (middle row), and precipitation rate (bottom row) of grid cells identified as regimes (a), (e) regime 1, (b), (f) regime 2, (c), (g) regime 4, and (d), (h) regime 6, for WQBO (orange) and EQBO (blue) composite days, in violin-style box-whisker plot (same convention as in Fig. 4). The conditions for compositing are simultaneous occurrence of MJO phases 6 and 7, and MJO amplitude in the 1-2 range during Non-El Niño winters. The significance of mean difference between WQBO and EQBO composites are obtained via a t-test, with the corresponding p-value shown at the top of each panel.

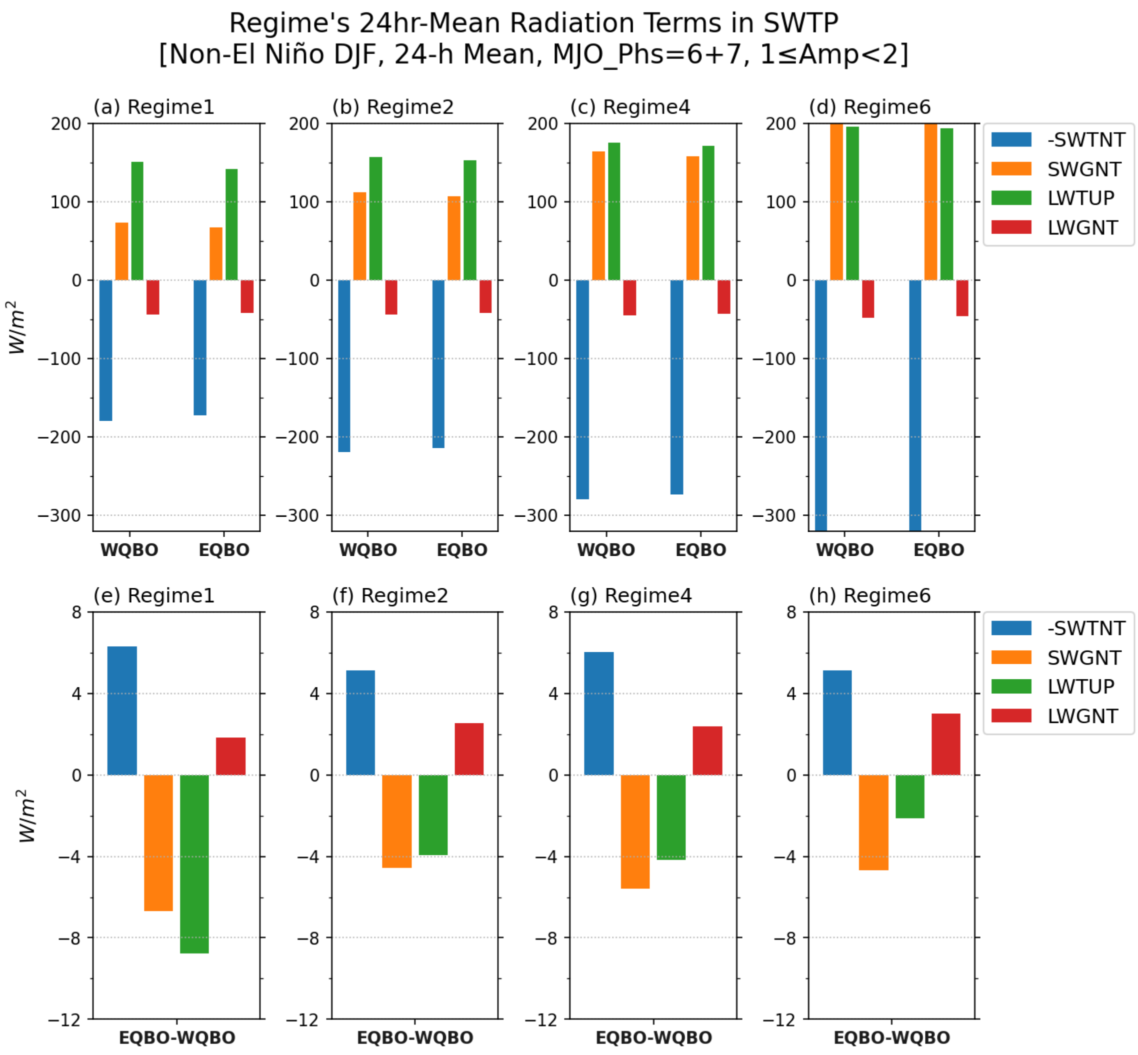

Figure S14. Comparison of radiative fluxes at the top of the atmosphere (TOA) and at the surface in deep convective systems within Madden-Julian oscillation (MJO) envelops between easterly Quasi-Biennial Oscillation (EQBO) and westerly QBO (WQBO) winters. (a)-(d) Distributions of southwestern tropical Pacific (SWTP; 150°E–160°W, 18°S–2°N) domain mean components of radiative flux divergence terms of grid cells identified as regimes (a) regime 1, (b) regime 2, (c) regime 4, and (d) regime 6, for WQBO and EQBO composite days. (e)-(h) Same as (a)-(d) but for the difference between WQBO and EQBO. The conditions for compositing are simultaneous occurrence of MJO phases 6 or 7, and MJO amplitude in the 1-2 range during Non-El Niño winters. "SWTNT" indicates net shortwave radiation at TOA, "SWGNT" net shortwave radiation at surface, "LWTUP" upward longwave radiation at TOA, and "LWGNT" net longwave radiation at surface. All terms are modified as positive meaning loss by atmospheric column.

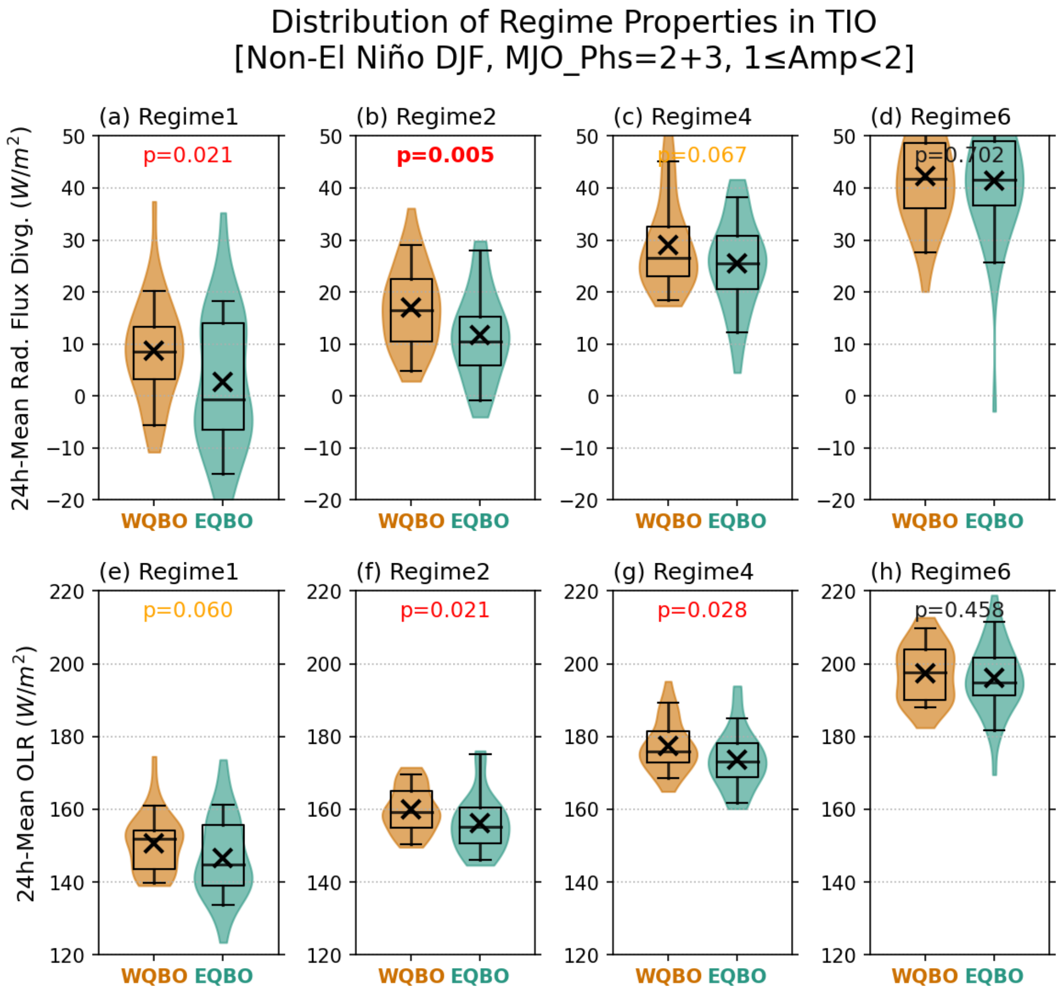

Figure S15. Comparison of column radiative flux convergence and outgoing longwave radiation (OLR) in deep convective systems within Madden-Julian oscillation (MJO) envelops between easterly Quasi-Biennial Oscillation (EQBO) and westerly QBO (WQBO) winters. Distributions of tropical Indian Ocean (TIO; 50°E–100°E, 12°S–8°N) domain mean cloud top pressure (top row) and brightness temperature (middle row), and precipitation rate (bottom row) of grid cells identified as regimes (a), (e) regime 1, (b), (f) regime 2, (c), (g) regime 4, and (d), (h) regime 6, for WQBO (orange) and EQBO (blue) composite days, in violin-style box-whisker plot (same convention as in Fig. 4). The conditions for compositing are simultaneous occurrence of MJO phases 2 and 3, and MJO amplitude in the 1-2 range during Non-El Niño winters. The significance of mean difference between WQBO and EQBO composites are obtained via a t-test, with the corresponding p-value shown at the top of each panel.

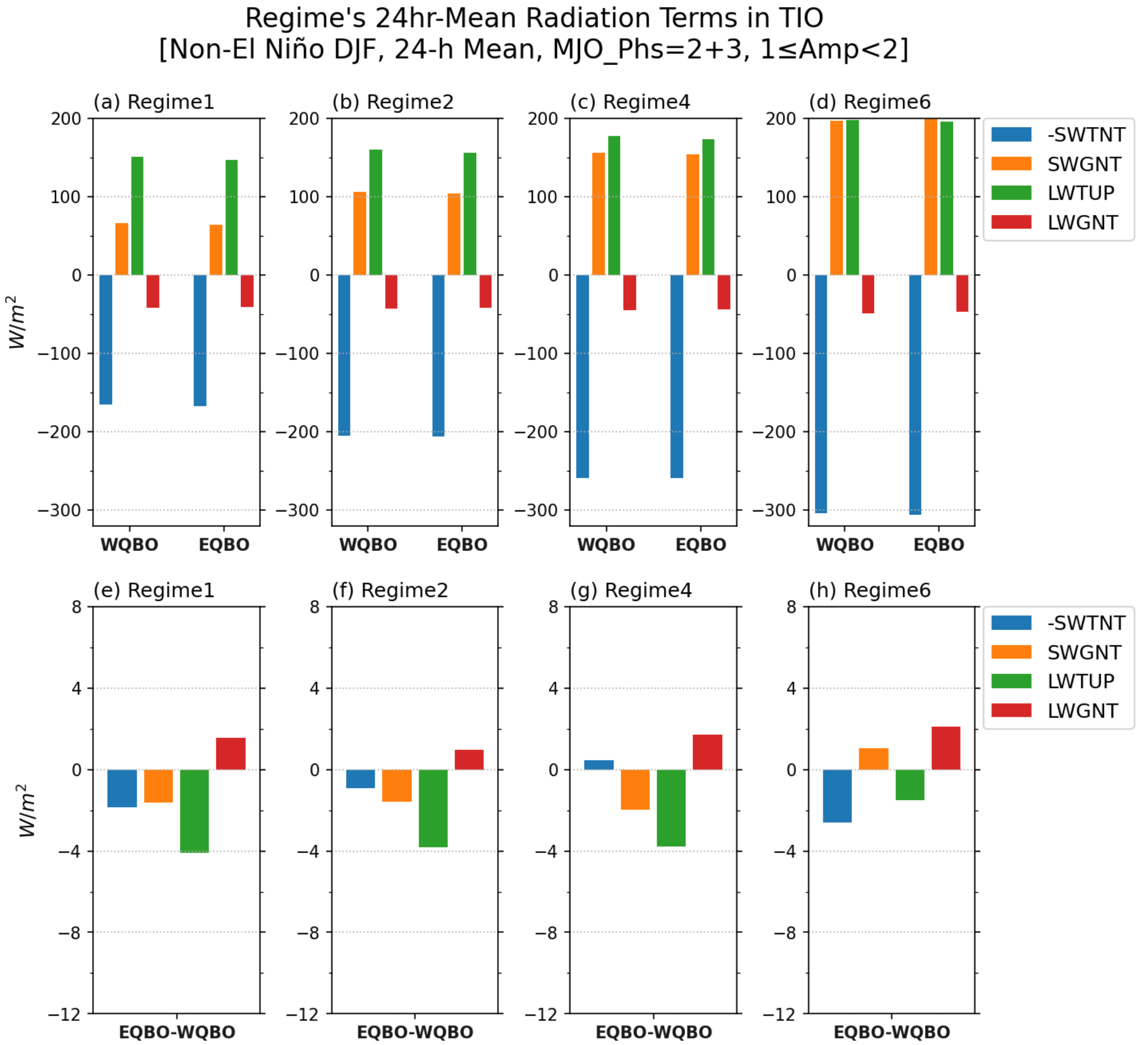

Figure S16. Comparison of radiative fluxes at the top of the atmosphere and at the surface in deep convective systems within Madden-Julian oscillation (MJO) envelops between easterly Quasi-Biennial Oscillation (EQBO) and westerly QBO (WQBO) winters. (a)-(d) Distributions of tropical Indian Ocean (TIO; 50°E–100°E, 12°S–8°N) domain mean components of radiative flux divergence terms of grid cells identified as regimes (a) regime 1, (b) regime 2, (c) regime 4, and (d) regime 6, for westerly Quasi-Biennial Oscillation (WQBO) and easterly QBO (EQBO) composite days. (e)-(h) Same as (a)-(d) but for the difference between WQBO and EQBO. The conditions for compositing are simultaneous occurrence of MJO phases 2 or 3, and MJO amplitude in the 1-2 range during Non-El Niño winters. “SWTNT” indicates net shortwave radiation at the top of the atmosphere (TOA), “SWGNT” net shortwave radiation at surface, “LWTUP” upward longwave radiation at TOA, and “LWGNT” net longwave radiation at surface. All terms are modified as positive meaning loss by atmospheric column.

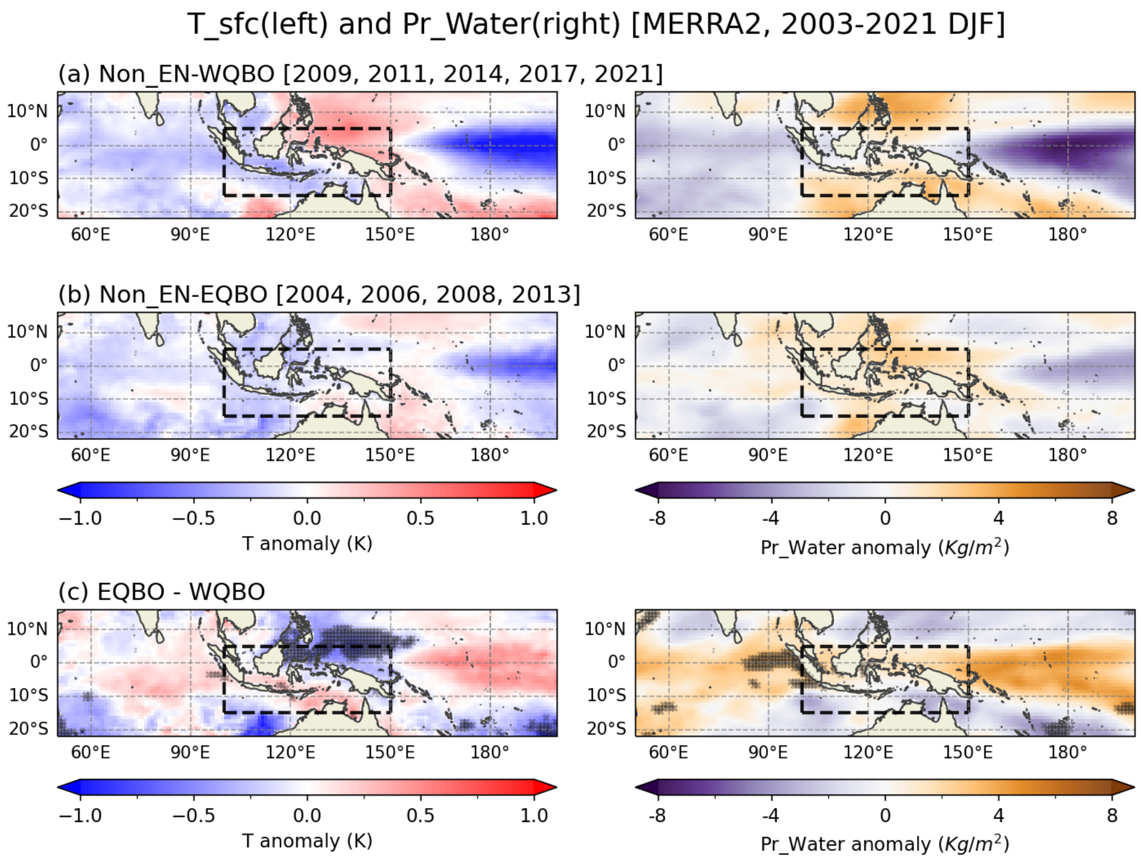

Figure S17. Quasi-Biennial Oscillation (QBO)-associated seasonal mean surface temperature and precipitable water anomalies. Seasonal (December to February) mean anomalies of surface temperature (left column) and precipitable water (right column) composited for the conditions of (a) Non-El Niño and westerly QBO (WQBO), (b) Non-El Niño and easterly QBO (EQBO), and (c) the difference between (a) and (b). The box in each panel outlines the boundaries of the MC domain (100°E–150°E, 15°S–5°N), and gray shading on bottom panels indicates the region of 95% significance level of t-test for each 5 and 4 seasons of WQBO and EQBO cases. The data over the period 2003-2021 is used.

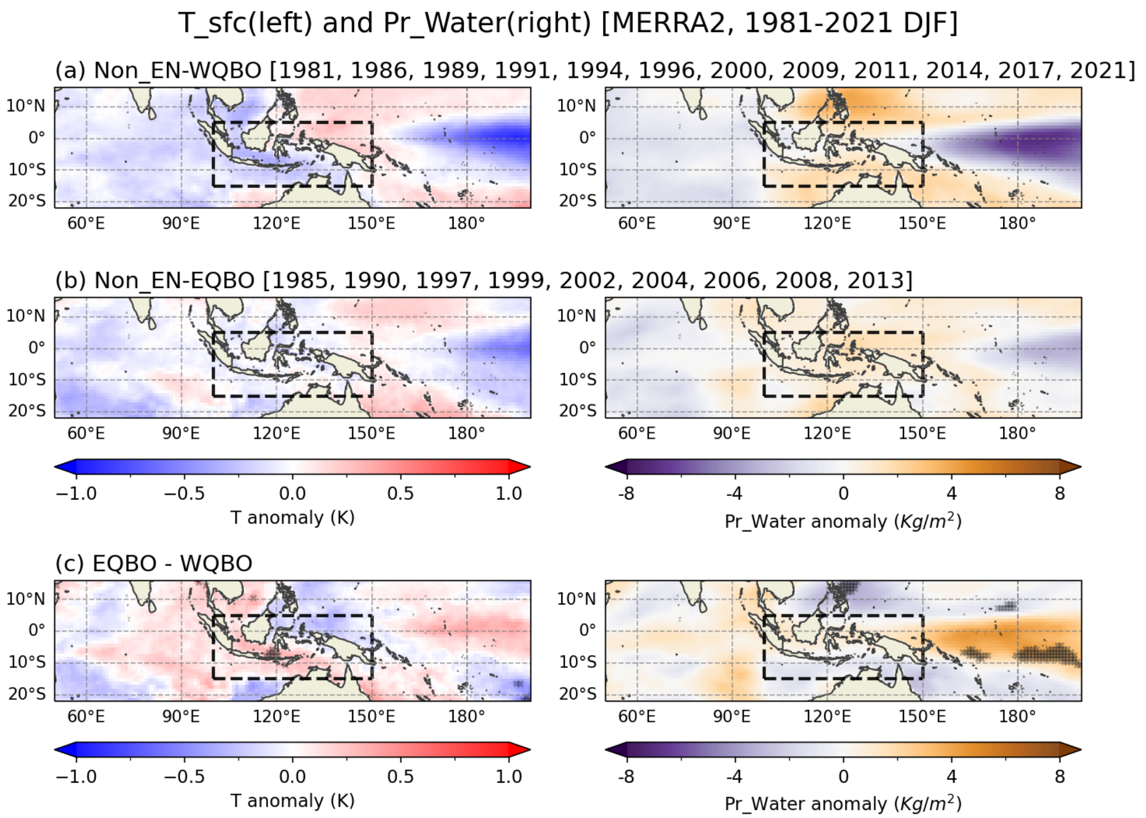

Figure S18. Quasi-Biennial Oscillation (QBO)-associated seasonal mean surface temperature and precipitable water anomalies. Seasonal (December to February) mean anomalies of surface temperature (left column) and precipitable water (right column) composited for the conditions of (a) Non-El Niño and westerly QBO (WQBO), (b) Non-El Niño and easterly QBO (EQBO), and (c) the difference between (a) and (b). The box in each panel outlines the boundaries of the MC domain (100°E–150°E, 15°S–5°N), and gray shading on bottom panels indicates the region of 95% significance level of t-test for each 5 and 4 seasons of WQBO and EQBO cases. The data over the period 1981-2021 is used.

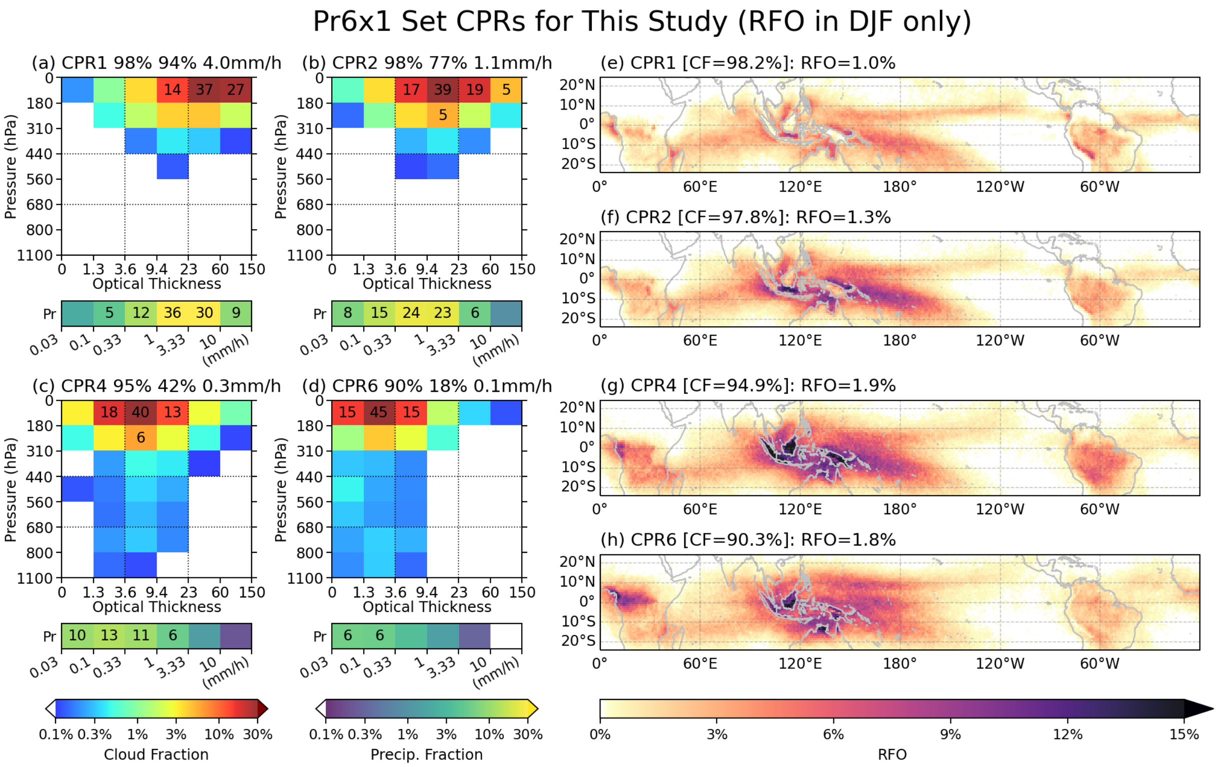

Figure S19. Definition of cloud-precipitation regimes. Select cloud-precipitation "hybrid" regimes (CPRs) derived in the deep tropical domain (15°S–15°N) from cloud and precipitation histograms that are naturally combined (referred to as Cld42+Pr6x1 set in Jin et al. 2021) in the clustering procedure. (a), (b), (c), and (d) Centroids of the cloud and precipitation components of CPR1, CPR2, CPR4, and CPR6, respectively. (e), (f), (g), and (h) Geographical distribution of the relative frequency of occurrence (RFO) of the CPRs from assignment of grid cell mean Terra-Aqua histograms in the boreal winter season (December to February).
